# A Tale of Two Features: Perception of Cantonese Lexical Tone and English Lexical Stress in Cantonese-English Bilinguals

**DOI:** 10.1371/journal.pone.0142896

**Published:** 2015-11-25

**Authors:** Xiuli Tong, Stephen Man Kit Lee, Meg Mei Ling Lee, Denis Burnham

**Affiliations:** 1 Division of Speech and Hearing Sciences, The University of Hong Kong, Hong Kong, SAR, China; 2 MARCS Institute, Western Sydney University, Sydney, New South Wales, Australia; UNLV, UNITED STATES

## Abstract

This study investigated the similarities and differences in perception of Cantonese tones and English stress patterns by Cantonese-English bilingual children, adults, and English monolingual adults. All three groups were asked to discriminate pairs of syllables that minimally differed in either Cantonese tone or in English stress. Bilingual children’s performance on tone perception was comparable to their performance on stress perception. By contrast, bilingual adults’ performance on tone perception was lower than their performance on stress perception, and there was a similar pattern in English monolingual adults. Bilingual adults tended to perform better than English monolingual adults on both the tone and stress perception tests. A significant correlation between tone perception and stress perception performance was found in bilingual children but not in bilingual adults. All three groups showed lower accuracy in the high rising-low rising contrast than any of the other 14 Cantonese tone contrasts. The acoustic analyses revealed that average F0, F0 onset, and F0 major slope were the critical acoustic correlates of Cantonese tones, whereas multiple acoustic correlates were salient in English stress, including average F0, spectral balance, duration and intensity. We argue that participants’ difficulty in perceiving high rising-low rising contrasts originated from the contrasts’ similarities in F0 onset and average F0; indeed the difference between their major slopes was the only cue with which to distinguish them. Acoustic-perceptual correlation analyses showed that although the average F0 and F0 onset were associated with tone perception performance in all three groups, F0 major slope was only associated with tone perception in the bilingual adult group. These results support a dynamic interactive account of suprasegmental speech perception by emphasizing the positive prosodic transfer between Cantonese tone and English stress, and the role that level of bilingual language experience and age play in shaping suprasegmental speech perception.

## Introduction

Most models of second language speech perception emphasize how differences in phonetic inventories—i.e., the specific vowels and consonants used in a language—affect bilinguals’ segmental speech perception [1–3]. However, languages also differ considerably in the way they use suprasegmental features, such as the use of lexical tone in Cantonese and the use of lexical stress in English to differentiate one word from another [4]. Cantonese lexical tones are pitch patterns used to minimally distinguish word meanings [5]. For example, the monosyllable /fu/ can represent six different meanings in Cantonese, each with a different tone: /fu55/膚(*skin*), /fu25/虎 (*tiger*), /fu33/褲 (*trousers*), /fu21/符 (*symbol*), /fu23/婦 (*woman*) and /fu22/父 (*father*) (The numerical notational system developed by Chao [6] is used to transcribe six different tones. The numbers 1 (lowest) to 5 (highest) are used to indicate relative height, shape and duration of pitch contour. High, middle and low indicate the relative average F0 while level, rising, falling specify the slope of the F0 contours). English lexical stress can also be used to convey differences in meaning; for example, PREsent/’pre-zənt/ (gift) versus preSENT/pri-‘zent/ (offer) (The capitalized letters represent stressed syllables). Like Cantonese tone, English stress uses pitch to convey such differences, but in contrast with tone, it only does so in conjunction with other acoustic parameters such as duration and intensity. These pitch-based differences, in single syllables in the case of tone and between contiguous syllables in the case of stress, have been largely unexplored in speech perception, especially for bilinguals of a tonal language (Cantonese) and a stress language (English). Thus, the goal of the present study is to examine similarities and differences in the perception of Cantonese tones and English stress patterns by Cantonese-English bilinguals, comparing bilingual children, bilingual adults, and English monolingual adults.

### Acoustic Correlates of Cantonese Lexical Tones and English Lexical Stress

**Fig 1:**
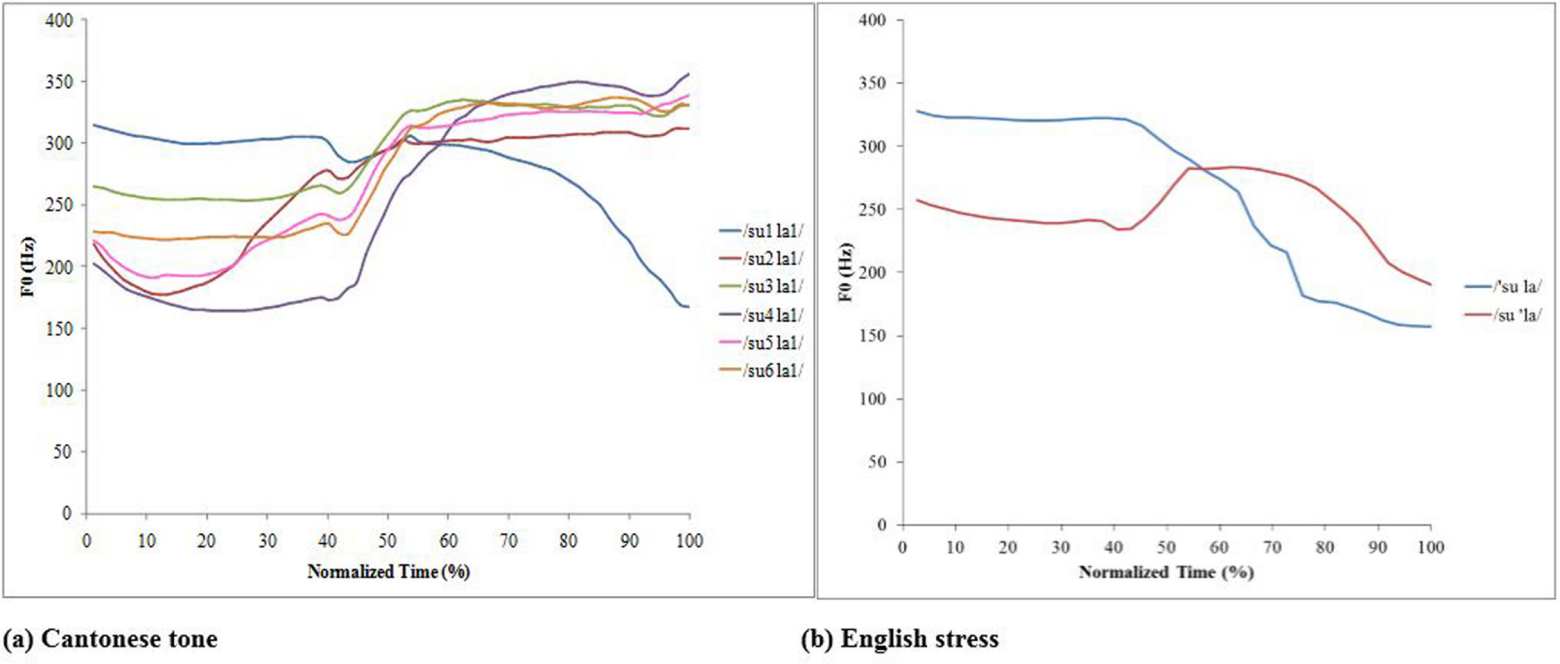
Normalized F0 traces of (a) six Cantonese tones and (b) two English stress patterns in the stimuli /sula/. Both tone and stress stimuli were produced by a bilingual female Cantonese speaker. /su1la1/ = high level (55), /su2 la/ = high rising (25), /su3la1/ = mid level (33), /su4la1/ = low falling (21), /su5la1/ = low rising (23), /su6la1/ = low level (22). /'sula/ = initial stressed syllable, /su'la/ = final stressed syllable.

The primary acoustic correlate of Cantonese tones is fundamental frequency (F0). As depicted in Fig 1A, the six Cantonese tones, i.e., high level (55, T1), high rising (25, T2), mid level (33, T3), low falling (21, T4), low rising (23, T5), and low level (22, T6) have several distinctive F0 characteristics, including height (i.e., the average F0 over time), contour (i.e., the change in the overall slope of F0 over time), and the values of F0 at onset and offset [5,7,8]. Here, T1 to T6 are the abbreviation terms for the six Cantonese tones.

The relative weighting of each of these acoustic features in Cantonese tone perception varies across different tone contrasts [9–11]. For example, Lee et al. showed that 3-year-old Cantonese children’s performance in discriminating the high rising (25, T2)-low falling (21, T4) tone contrast was significantly lower than their performance in discriminating high level (55, T1)-high rising (25, T2) and high level (55, T1)-low falling (21, T4) tone contrasts [10]. Notably, T2 and T4 have similar F0 onset values but different F0 offset values, whereas the tone contrasts that children were better able to discriminate (i.e., T1-T2 and T1-T4) have large differences in F0 onset. The authors concluded that the similarity of two tones’ F0 onset plays a much more important role than F0 offset similarity in predicting the relative ease with which tone contrasts can be perceived. Tong and colleagues extended this line of work by showing that 5- to 6-year-old Cantonese children attended to F0 onset for the perception of similarly-contoured tones such as high rising (25, T2)-low rising (23, T5), while using F0 contour to distinguish tones with different contours such as high level (55, T1)-high rising (25, T5) [11].

Similarly to Cantonese tone, English lexical stress involves modulation in pitch. As shown in Fig 1B, stressed syllables tend to have a higher average F0 compared to unstressed syllables. However, unlike Cantonese tones, which use F0 alone to convey phonetic distinctions, the realization of English stress depends on F0 as well as other acoustic features such as duration, intensity, spectral balance (the distribution of intensity over different frequency bands) and vowel quality [12–16].

There is perceptual evidence showing that some of these acoustic parameters—specifically, F0, duration, and intensity—appear to be more important than others in distinguishing stressed syllables from unstressed ones [15,17,18]. For example, Fry found that among the three acoustic correlates of English lexical stress, i.e., average F0, duration, and intensity, F0 is the strongest cue in perceiving English lexical stress, followed by syllable duration and intensity [18]. Bolinger also suggested that average F0 is the primary cue for English lexical stress perception, whereas syllable duration and intensity are secondary cues [17].

Taken together, these findings suggest that the perception of Cantonese tone and English lexical stress rely on some similar acoustic concomitants. This raises the possibility that certain acoustic cues, such as F0, may be employed by Cantonese-English bilinguals to perceive both Cantonese tones and English stress patterns. Given that these previous findings are derived exclusively from empirical studies of monolingual speakers of Cantonese and English, there is a remarkable lack of evidence addressing this question in bilingual speakers. Empirical work is needed to determine how different acoustic correlates—including F0 onset, average F0, F0 contour, duration, intensity, and spectral balance—are employed by Cantonese-English bilingual speakers to perceive Cantonese tones and English stress patterns, and furthermore to identify the relationships between bilinguals’ sensitivity to acoustic cues and their ability to perceive tones and stress patterns. Thus, the first aim of the present study is to examine Cantonese-English bilingual speakers’ relative use of different tone- and/or stress-related acoustic correlates in perceiving Cantonese tones and English stress patterns, compared to the use of these cues by English monolinguals. We also examine the relationships between bilinguals’ sensitivity to acoustic cues and their performance in perceiving tone and stress contrasts.

### Cross-Language Perception of Cantonese Lexical Tones and English Lexical Stress

There is growing evidence that native language experience influences the perception of non-native lexical tone [19–23]. For example, Gandour et al. demonstrated that native adult speakers of a tonal language (Thai) exhibited brain activation in the left frontal operculum (a region near Broca’s area) when discriminating pitch patterns in Thai words [20]. However, there was no such brain activation in speakers of a non-tonal language (English). In a perceptual learning study with native adult speakers of tonal Mandarin Chinese or non-tonal English, Francis et al. showed that the Mandarin speakers were better at learning and identifying low level (22, T6), low falling (21, T4) and low rising (23, T5) Cantonese tones than the English speakers, suggesting that one’s native language experience with suprasegmental or prosodic structure affects the learnability of Cantonese lexical tones [19].

Further support for this claim comes from So and Best [21], who provided extensive analyses of non-native Mandarin lexical tone perception by adult speakers of Cantonese (a tonal language), Japanese (a pitch accent language) and English (a stress accent language). They found that Cantonese speakers successfully perceived high level (55)-mid rising (35) Mandarin tone contrasts, but exhibited difficulty in perceiving high level (55)-high falling (51) and mid rising (35)-falling rising (214) tone contrasts. On the other hand, the Japanese and English speakers did not have this difficulty. The authors explained that the tone confusion pattern exhibited by Cantonese speakers reflects the already existing phonetic similarities between the Cantonese and Mandarin tonal systems. They also found that overall tone perception accuracy was higher among the Japanese speakers than the English speakers, a finding that can be attributed to the fact that Japanese’s pitch-accent (mora) characteristics are more acoustically and functionally similar to Mandarin lexical tones than English lexical stress is.

Likewise, there are a growing number of studies examining non-native stress perception and production that suggest that listeners’ native language experience with prosodic information influences their perception and acquisition of English lexical stress. For example, Archibald found that Polish speakers were more likely to misplace stress on English words with a heavy final syllable such as mainTAIN, apPEAR, or deCIDE (e.g., producing MAINtain) [24], because in Polish, stress always falls on the penultimate syllable. In related work, Archibald demonstrated that Hungarian speakers experienced difficulties in assigning primary stress to English words with consonant-vowel-consonant (CVC) structure such as colLAPSE, eLECT, or obSERVE [25], because stress is usually placed on the word-initial syllable in Hungarian. Similarly, Spanish speakers were found to have difficulties in perceiving initially stressed English words such as CENtral, BAsis, or REAson, and also in producing words with antepenultimate stress such as ANecdote, INterface, and UNdertow, because they violate Spanish stress rules—in Spanish, the stress of a word is placed on any one of the final three syllables [26].

Similar effects of an individual’s native language on non-native English stress assignment have also been reported in tone language speakers [16,27]. For example, Zhang et al. provided extensive analyses concerning the acoustic correlates of English lexical stress produced by native Mandarin speakers [16]. They found that Mandarin speakers tended to produce stressed English syllables with a higher average F0 than did American English speakers, and that the Mandarin speakers, but not the English speakers, exhibited a significant difference in F0 peak timing between stressed syllables and unstressed syllables. Moreover, the Mandarin speakers failed to produce reduced vowels in unstressed syllables. The authors concluded that “Mandarin speakers’ production of lexical stress contrasts in English is influenced partly by native-language experience with Mandarin lexical tones, and partly by similarities and differences between Mandarin and English vowel inventories” ([16], pp.4498). Similarly, Chan showed that experience with tonal Cantonese influences the use of acoustic cues in non-native English stress perception [27]. Specifically, native Cantonese speakers used F0 as the primary acoustic cue to perceive English stress patterns, whereas English speakers used spectral balance as the primary cue.

Collectively, findings from these studies on cross-language perception of suprasegmental speech contrasts have alluded to the possibility that Cantonese lexical tone and English lexical stress are processed interactively, at least in the very early stage of acoustic-phonetic processing, because they involve processing some of the same acoustic features, such as fundamental frequency (F0). This interdependent and interactive processing of suprasegmental speech features may be even more evident for Cantonese-English bilinguals who speak one language that employs tones (Cantonese) and another language that employs stress patterns (English), as these bilinguals may build a holistic, common representation of suprasegmental features for their two languages. Thus, the second aim of the present study is to explore this possibility by examining the similarities and differences between Cantonese tone perception and English stress perception in Cantonese-English bilinguals.

These previous studies pointing to the effects of one’s native language on non-native suprasegmental speech perception are almost exclusively done with skilled adults. However, a very recent study by Quan & Swingley has shown that age affects native English lexical stress perception among monolingual English speakers [28]; specifically, young children's English lexical stress perception was worse than skilled English-speaking adults. The authors argued that adults had far more language experience than children, leading to differences between the groups in English stress perception. There is also compelling evidence showing auditory capabilities mature with age, with children using different acoustic cues than adults in auditory processing tasks [29,30]. Findings from these previous studies suggest that children and adults have different levels of linguistic knowledge about their languages and that children have different phonological processing capabilities than adults. Thus, from a theoretical viewpoint, a comparison between bilingual children and adults in the perception of Cantonese tones and English stress patterns sheds new light on the role of linguistic knowledge in the perception of suprasegmental speech. Furthermore, studying differences in the use of acoustic cues by bilingual children and bilingual adults offers insights into the acoustic cues underlying the perception of Cantonese tone and English stress across different ages of bilinguals. Given that no studies to date have examined or compared the similarities and differences between developing bilingual children and skilled bilingual adults in perceiving Cantonese tones and English stress patterns, the third aim of the present study is to determine the effect of linguistic knowledge on Cantonese tone and English stress perception by comparing children with skilled adults.

### The Present Study

An overarching goal of the present study is to test a dynamic interactive theory of suprasegmental speech processing in bilinguals. This theory would argue that Cantonese-English bilinguals process tone and stress interactively in a way that varies as a function of the development of their linguistic knowledge of each language’s prosodic system. The dynamic interactive theory of suprasegmental speech processing incorporates both between-language and within-language interactions by assuming an interaction between tone and stress, and an interaction between top-down (unconscious expectation) and bottom-up (acoustic analysis of the stimuli) processes within each language. These interactions evolve and develop over an extended period of time, and therefore are expected to vary as a function of developmental differences in bilinguals’ linguistic knowledge of the two prosodic systems. To test this account, the current study evaluates the differences and similarities between perception of tone and stress in Cantonese-English bilingual children and adults, as well as English monolingual adults. There are three related but distinct hypotheses relevant to this account. The first hypothesis is that if tone and stress are interdependently and interactively processed, there will be a significant positive correlation between Cantonese tone perception and English stress perception in both bilinguals and monolinguals. This hypothesis rests on the acoustic and perceptual evidence showing that Cantonese tone and English stress share similar acoustic concomitants [8,18], and that the perception of tone and stress involve processing of the same acoustic feature, i.e., F0 [16,27].

The second hypothesis is that if tone and stress are interactively and dynamically processed, we will find differences between bilingual children and adults in their perception of Cantonese tone and English stress due to age-related differences in their acoustic-phonetic processing capabilities. Given their more extensive L1 and L2 exposure, bilingual adults have much richer experience in the use of Cantonese tone and English stress than bilingual children do, and their understanding of the languages’ tone and stress systems is much more sophisticated and fine-grained than that of bilingual children. They can generate expectations about acoustic cues that are crucial to distinguishing tone and stress contrasts much more quickly than bilingual children, and they can make more efficient use of acoustic features to verify those expectations. Thus, the interactive processing of tone and stress is much faster and more efficient in bilingual adults than in bilingual children, and the interaction between top-down expectations and bottom-up acoustic analyses is likewise more efficient. Conversely, due to their developmental immaturity and their less extensive linguistic knowledge of L1 and L2, bilingual children may not preserve fine-grained acoustic details of Cantonese tone and English stress. Thus, they may not yet have developed subtle sensitivity to the acoustic cues that distinguish tone and stress contrasts, particularly less dominant cues (such as major slope for lexical tone). This would be evidenced by differences in both tone perception and stress perception performance, and also in the relative use of different acoustic cues to distinguish difficult tone contrasts, such as high rising (25)-low rising (23).

The third hypothesis is that if Cantonese tones and English stress patterns are processed interactively, we would expect prosodic transfer between Cantonese tones and English stress in Cantonese-English bilinguals but not in English monolinguals. That is, we would expect that Cantonese-English bilinguals, at least bilingual adults, will show better performance in perceiving Cantonese tones and English stress patterns than English monolingual adults, because under the dynamic interactive account, tones and stress would be able to mutually facilitate each other during the course of suprasegmental processing. Moreover, we would expect our three participant groups (both bilinguals and monolinguals) to use acoustic correlates common to tones and stress, such as F0, in perceiving both Cantonese tones and English stress, and we would expect significant positive associations between F0 acoustic correlates and tone perception in the three groups.

These three hypotheses were examined with an AXB perceptual experiment, as well as an extensive analysis of all acoustic correlates of the large set of Cantonese tones and English stress stimuli used in our perceptual experiment. Most previous studies on suprasegmental speech perception have used a relatively small number of perceptual stimuli, and as such have lacked the type of systematic acoustic analyses that might shed light on the acoustic features that underlie the perception of suprasegmental speech features. By including a large set of suprasegmental stimuli and carrying out such analyses in the current study, we will be better able to draw detailed conclusions about the processes by which Cantonese-English bilinguals’ perceive the suprasegmental features of speech. By testing these hypotheses in Cantonese-English bilinguals for whom one language employs lexical tone (Cantonese) and the other employs lexical stress (English), we are able to examine the extent to which one’s native language experience (e.g., to a tonal language) influences the perception of suprasegmental features of another language (e.g., stress). This will help us to better understand the cross-language representation and transfer of prosodic features.

## Method

### Participants

Three groups of participants were included in the present study: 30 Cantonese-English bilingual children (20 girls, 10 boys; mean age = 11.70 yrs, *SD* = 2.28 yrs), 30 Cantonese-English bilingual adults (11 female, 19 male; mean age = 21.06 yrs, *SD* = 1.22 yrs); and 15 English monolingual adults (13 female, 2 male; mean age = 24 yrs, *SD* = 4.54 yrs). Eleven-year-old children were selected for this study because of clear evidence showing that children at this age perform as well as adults in perceiving Cantonese tones [31] and English lexical stress [32]. Cantonese-English bilingual children were recruited from Hong Kong International Primary Schools or English-medium schools. Based on the parent-report questionnaire data, the children included in our study were all from families with a medium to high income level (the monthly family income range in Hong Kong is 3,867–6,445 USD according to the Hong Kong Census and Statistics Department [33]). All children’s mothers had attained education at a bachelor’s level or above. The Cantonese-English bilingual adults in our study were all undergraduate students at the University of Hong Kong, an English-medium university, and the English monolingual adults were either university students or staff in Hong Kong. We obtained written informed consent from all adult participants and from the parents of all participating children. The research protocol was approved by the Human Research Ethics Committee for Non-Clinical Faculties of The University of Hong Kong (EA040711).

Participants’ bilingual or monolingual status was assessed with a revised Language, Social and Music Background Questionnaire that was adopted from Bialystok and Depape [34]. The questionnaire was administered to the parents of participating children (parent-report) and the adult participants (self-report) prior to testing, and it collected information about the participant’s first and second language exposure, level of fluency in both languages, and home literacy practices. The questionnaire also gathered information about the participant’s parents’ occupation, income, and education level, which was then indexed as the participant’s socioeconomic status. The children’s parents and the adult participants were asked to rate their oral and aural ability in Cantonese and English on a 5-point scale ranging from poor (1) to excellent (5). Our analyses of parents’ reported data showed that the mean percentage of time that their child spoke in English and Cantonese each day was 53.17% and 46.83%, respectively. Parents’ ratings of spoken language proficiency suggested that the Cantonese-English bilingual children’s Cantonese spoken proficiency (*M* = 3.80, *SD* = 1.32) was not statistically distinguishable from their English spoken proficiency (*M* = 4.37, *SD* = 0.72), *t*(29) = -0.46, *p* = .65. Likewise, parents’ ratings of children’s Cantonese listening comprehension (*M* = 4.03, *SD* = 0.93) were comparable to their ratings of English listening comprehension (M = 4.33, SD = 0.80), *t*(29) = -0.19, *p* = .85.

By contrast, adult Cantonese-English bilingual participants reported speaking in English about 12.37% of the time each day and in Cantonese about 87.63% of the time. They rated their Cantonese spoken proficiency (*M* = 4.67, *SD* = 0.48) higher than their spoken proficiency in English (*M* = 3.43, *SD* = 0.97), *t* (29) = 5.40, *p* < .001 and rated their Cantonese listening comprehension (*M* = 4.73, *SD* = 0.52) as being higher than their English listening comprehension (*M* = 3.63, *SD* = 0.93), *t* (29) = 4.94, *p* < .001.

Parents reported that Cantonese-English bilingual children start learning English at birth. Cantonese-English bilingual adults reported that they started learning English at 3.73 years old (*SD* = 2.16 yrs). English monolingual adults reported English as their native language, and they had no experience learning Chinese or any other tone language.

According to parents’ reports and adults’ self-report, our participants had no history of hearing, language and learning difficulties. All of them passed a pure-tone hearing screen (250, 500, 1000, 2000, 4000HZ at 20dB SPL).

### Materials

Fifty-five pseudowords were constructed as stimuli (for examples, see Fig 1A and 1B). These pseudowords complied with the phonotactic rules of both Cantonese and English, but none of them carried any lexical meanings. The use of pseudowords eliminates any influence of the properties of lexicons and intrinsic knowledge of pitch, loudness and duration of vowels of real words on the perception of linguistic pitch [15]. All of the pseudowords were disyllabic with each syllable bearing a Consonant-Vowel (CV) structure. The CV syllable was used because it is the core syllable in both Cantonese and English, and it is universal in all languages [35–37]. The CVCV structure has also been successfully used to assess the perception of suprasegmental features of speech in different languages [16,27,38].

The 55 pseudowords were created by using 11 consonants (/b/, /p/, /d/, /t/, /g/, /k/, /m/, /h/, /l/, /s/, and /f/) and five vowels (/a/, /ɛ/, /i/, /ɔ/, and /u/). There are three theoretical reasons for selecting these consonants and vowels. First, they have similar phonetic realization in both Cantonese and English, and they are commonly used in both languages [7,39]. Thus, the use of these consonants and vowels can control for the familiarity of phonetic segments across languages, which recent research has shown to influence perception of suprasegmental contrasts [11]. Second, the eleven consonants cover five different places of articulation, (i.e., bilabial /b/, /p/ and /m/, labial-dental /f/, alveolar /d/, /t/, /s/ and /l/, velar /g/, /k/ and glottal /h/), as well as four manners of articulation (i.e., plosive /b/, /p/, /d/, /t/, /g/ and /k/, fricative /f/, /s/ and /h/, nasal /m/, and lateral approximant /l/), and the five vowels selected are commonly used in both Cantonese and English. The combination of these consonants and vowels can create large sets of stimuli containing most of the possible acoustic variability existing in lexical tone and stress perception outside the laboratory. Third, language acquisition research has shown that the acquisition order of these consonants is the same between Cantonese and English, despite differences in acquisition rate [40,41]. Furthermore, according to the norms in the Hong Kong Cantonese Articulation Test, these consonants are all acquired before school age. Thus, the use of these consonants and vowels eliminates the possible influences of the acquisition order of phonetic segments on perception of suprasegmental features.

The consonants and vowels were assigned randomly into CVCV structures to form disyllabic pseudowords (e.g., /sula/, /hɔmi/, /lamu/), with the restriction that none of the CVCV structures were allowed to contain any repeating consonants or vowels. These pseudowords were used to carry Cantonese lexical tones and English lexical stress.

#### Cantonese tone perception test

There were two types of CVCV Cantonese stimuli; they consisted of the same phonetic segments and differed only in the location of constant tone. In the first type, the tone of the first syllable remained constant, i.e., high level tone (55, T1), while the second syllable carried six tones, varying from tone 1 to 6 (see Fig 1A). In the second type, the tone of the first syllable varied from tone 1 to 6, while the second syllable carried constant tone. In both cases, the high level tone (55, T1) was used as the constant tone because, firstly, it has the highest occurrence in Cantonese [42]; both the speakers recording pseudowords and the participants should therefore be most familiar with this tone. Secondly, Hong Kong Cantonese speakers find tone contrasts with high level tones easier to distinguish, as the high level tone is well separated from the other five tones in terms of its pitch value [43,44]. Therefore, it should exert the least influence on production in the stimulus recordings and the participants’ perception in the experiment.

A total of 15 tone contrasts were formed by pairing the six distinct Cantonese tones: T1-T2, T1-T3, T1-T4, T1-T5, T1-T6, T2-T3, T2-T4, T2-T5, T2-T6, T3-T4, T3-T5, T3-T6, T4-T5, T4-T6, and T5-T6. These 15 tone contrasts were carried by our 55 CVCV pseudoword sequences. A balanced latin-square design was used to control for the variation of tone contrasts and CVCV structures. The 15 tone contrasts and 55 CVCV sequences were both randomly grouped into five blocks, with each block comprising 11 CVCV sequences carrying three tone contrasts. With this design, we generated five versions of the stimulus set, each having 165 trials (3 tone contrasts × 11 CVCV sequences × 5 blocks). This design enabled the inclusion of all 55 CVCV stimuli and all 15 tone contrasts in each version. In addition, to counterbalance the order of the position of the constant tone, two types of Cantonese stimuli (1^st^ syllable with the constant tone; 2^nd^ syllable with the constant tone) were used. Thus, a total of 10 versions of the Cantonese stimuli were used for the tone perception test.

#### English stress perception test

The same 55 CVCV sequences were used to construct English stress stimuli (see Fig 1B). Stress was placed on either the first or the second syllable, producing initial-stressed (IS) and final-stressed (FS) pseudowords, respectively. This yielded total of 110 stimuli (55 CVCV × 2 stressed conditions). An example of six Cantonese tones and two English stress patterns that were carried by the CVCV sequence /sula/ is shown in Fig 1A and 1B, respectively.

### Recording and Segmentation

The items carrying both Cantonese tones and English stress were recorded three times each by a female bilingual who had received phonetic training and had a linguistic background in both Cantonese and English. She was born in Hong Kong, but lived in an English-speaking country for 10 years, and her Cantonese and English had both reached native-like levels of proficiency (according to ratings of her production of Cantonese and English words by native speakers of Cantonese and English, respectively). She was informed about the background of the experiment and was aware that the items recorded would be used as stimuli in the experiment.

All utterances were recorded in a soundproof booth and were digitized at a sampling rate of 44.1 kHz (16 bit, mono) using the phonetic analysis software Praat [45]. The tone and stress stimuli were written on slides and presented to the speaker separately, with each slide showing one written stimulus in IPA transcription. The speaker was asked to produce each stimulus three times, and was asked to count ‘1, 2’ after each stimulus to confirm that she was ready for the next stimulus. The pace of the slide shows was controlled by the experimenter. Tone and stress stimuli were recorded separately, while the order of stimuli within each recording session was randomized. Practice trials were provided before recording began. The speaker was asked to produce the stimuli at a tone level she was comfortable with. A total of 2310 stimuli were recorded (55 CVCV × 6 tones × 2 types × 3 repetitions + 55 CVCV × 2 stress conditions × 3 repetitions = 1980 + 330 = 2310). The recordings were made over two separate days.

All recordings were edited using Praat [45]. We excluded all recorded stimuli that contained disfluencies, that were unclear or had unstable loudness, or that were of extreme durations (longer than 1000ms or shorter than 700ms). All segmentations were made at zero crossings, as determined auditorily and by visual inspection of waveforms and spectrograms. The selected stimuli for both the Cantonese tone and English stress perception tests were between 800ms and 1000ms, and the intensity of all recordings were between 50dB to 66dB. A total of 660 (55 CVCV × 2 types × 6 tones) tone stimuli and 110 (55 CVCV × 2 stress conditions) stress stimuli were selected for the perception tests.

### Procedure

The experiment was conducted in a soundproof room at the University of Hong Kong. We used the AXB discrimination paradigm that has been widely used in previous research on speech perception with both adults and children [46–48]. A trial consisted of three auditory stimuli (with either Cantonese tone or English stress) presented sequentially to participants, separated by 400ms. In each trial, either the first (e.g., tone trial: /ku1ma5/, /ku1ma5/, /ku1ma2/; stress trial: /kuˈma/, /kuˈma/, /ˈkuma/) or the third stimulus (e.g., tone trial: /la1mu2/, /la1mu4/, /la1mu4/; stress trial: /laˈmu/, /ˈlamu/, /ˈlamu/) in the sequences shared the same tone/stress pattern as the second stimulus. The participants were asked to decide whether the second stimulus sounded more like (or the same as) the first or the last stimulus within 3000ms by pressing the “1” or “3” button on the keyboard, respectively. A new trial automatically started if the participants did not make any response within 3000ms [49,50]. There were four AXB orders (AAB, ABB, BAA, BBA) in terms of target position and choice position. In order to avoid any bias due to choice positions, these four orders of choice and target arrangement were randomly assigned to the stimuli. Testing began with practice trials, followed immediately by the test trials. The inter-trial interval was 400ms. We used 3000ms as the interstimuli interval and 400ms as the inter-trial interval because the auditory span was 200-300ms and the processing time of 250ms should be sufficient for recognizing a speech signal [51].

All stimuli were played automatically in a randomized order by the Java program on an IBM Thinkpad Lenovo laptop computer. Both Cantonese tone and English stress stimuli were set to have average level of 63dB SPL. The participants listened to the stimuli via headphones connected to the computer. Accuracy and reaction time were automatically recorded by the program. The Cantonese tone and English stress perception tests each took 10–15 minutes, and a break was given between the two sessions.

## Results

One lexical tone stimulus (LUki4) was excluded from all the analyses due to experimental manipulation error. Preliminary data analyses of the lexical tone perception task showed no significant main effect of the position of the constant tone (1^st^ syllable vs. 2^nd^ syllable) for either accuracy, *F*(1, 73) = 0.00, *p* = .995 or response latency, *F*(1, 73) = 1.42, *p* = .238. Thus, the two types of Cantonese tone stimuli were collapsed in the following data analyses.

### Differences in Perception of Cantonese Tone and English Stress by the Three Groups

**Fig 2:**
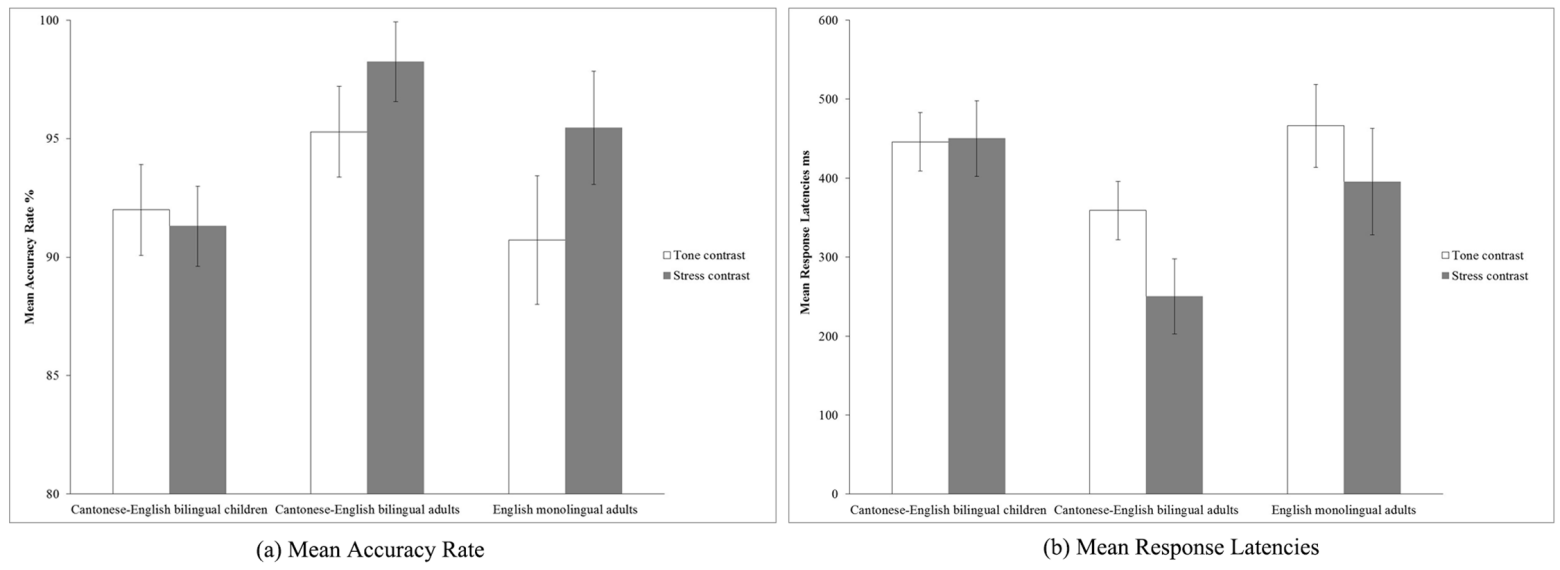
(a) Means showing proportion of correct responses (i.e. accuracy rates) for the three groups of participants toward Cantonese tone contrasts and English stress contrasts. Error bar represents 95% confidence intervals. (b) Means showing mean response latencies (i.e., reaction times) for the three groups of participants toward Cantonese tone contrasts and English stress contrasts. Error bar represents 95% confidence intervals.

The three participant groups’ mean proportion of correct responses (i.e., accuracy rates) and mean response latencies (i.e., reaction times) for Cantonese tone contrasts and English stress contrasts are shown in Fig 2A and 2B, respectively. The mean response latencies reported here are based on items that were responded to correctly. We excluded trials on which participants made extremely fast or slow responses (i.e., more than 2SDs above or below the mean per condition per subject) from our latency analyses; this amounted to less than 5% of all trials.

The mean accuracy rates for tone contrasts and stress contrasts were examined in a two-way analysis of variance (ANOVA) with pitch contrast (Cantonese tone vs. English stress) as a within-subjects factor and group (Cantonese-English bilingual children, Cantonese-English bilingual adults, English monolingual adults) as a between-subjects factor. There were significant main effects of pitch contrast, *F*(1, 72) = 19.12, *p* < .001, η^2^_p_ = .21, and group, *F*(2, 72) = 10.28, *p* < .001, η^2^_p_ = .22. The crucial result was a significant interaction between pitch contrast and group, *F*(2, 72) = 9.24, *p* < .001, η^2^_p_ = .20.

As shown in Fig 2A, the Cantonese-English bilingual children’s performance on Cantonese tone perception (*M* = .92 correct) was statistically indistinguishable from their performance on English stress perception (*M* = .91 correct), *t*(29) = 0.94, *p* = .355. However, the adult Cantonese-English bilinguals’ performance on Cantonese tone perception (*M* = .95 correct) was significantly lower than their performance on English stress perception (*M* = .98 correct), *t*(29) = -3.45, *p <* .01. Similarly, the adult English monolingual group’s performance on Cantonese tone perception (*M* = .90 correct) was significantly lower than their performance on English stress perception (*M* = .95 correct), *t*(14) = -4.15, *p <* .01.

Meanwhile, the simple main effect of group was also significant for both tone perception, *F*(2, 72) = 4.76, *p* < .05, η^2^_p_ = .12, and stress perception, *F*(2, 72) = 16.95, *p* < .001, η^2^_p_ = .32. A Fisher’s least significant difference (LSD) post hoc test revealed that Cantonese-English bilingual adults had significantly higher accuracy rates than Cantonese-English bilingual children on tone (*p* < .05) and stress (*p* < .001) perception. Cantonese-English bilingual adults performed better than English monolingual adults at tone perception (*p* < .01), and their performance on stress perception was marginally higher than English monolingual adults (*p* = .061). Cantonese-English bilingual children had lower accuracy on stress perception than English monolingual adults (*p* < .05), with no difference in tone perception (*p* = .446).

The mean response latencies for Cantonese tone contrasts and English stress contrasts were submitted to a similar two-way ANOVA. There were significant main effects of pitch contrast, *F*(1, 72) = 19.17, *p* < .001, η^2^_p_ = .21, and group, *F*(2, 72) = 15.98, *p* < .001, η^2^_p_ = .31. The interaction between pitch contrast and group was also significant, *F*(2, 72) = 8.13, *p* < .01, η^2^_p_ = .18.

As shown in Fig 2B, further analysis of the interaction effect showed that Cantonese-English bilingual children’s response latencies to Cantonese tone contrasts (*M* = 446ms) were statistically indistinguishable from their latencies to English stress contrasts (*M* = 450ms), *t*(29) = -.19, *p* = .848. However, Cantonese-English bilingual adults showed significantly longer response latencies toward Cantonese tone contrasts (*M* = 359ms) than English stress contrasts (*M* = 250ms), *t*(29) = 7.48, *p <* .001. Similarly, English monolingual adults tended to show slower responses to Cantonese tone contrasts (*M* = 466ms) than to English stress contrasts (*M* = 396ms), *p* = .070. Additionally, a Fisher’s LSD post hoc test revealed that Cantonese-English adults responded more quickly than Cantonese-English bilingual children on the tone perception (*p* < .01), and stress perception (*p* < .001) tests. Similarly, bilingual adults responded more quickly than English monolingual adults on both the tone perception (*p* < .01), and stress perception (*p* < .01) tests. However, there was no difference between the response latencies of bilingual children and English monolingual adults on either the tone perception test (*p* = .530) or the stress perception test (*p* = .192).

Next, we conducted correlational analyses on the accuracy of Cantonese tone and English stress perception, exploring their association separately for each group. We found a significant correlation between Cantonese tone perception and English stress perception accuracy in Cantonese-English bilingual children (*r* = .73, *p* < .001) and English monolingual adults (*r* = .79, *p* < .01). However, there was no significant correlation between tone perception and stress perception accuracy in Cantonese-English bilingual adults (*r* = .13, *p* = .49).

### Perceptual Saliency of Different Tone Contrasts Across Groups

**Fig 3:**
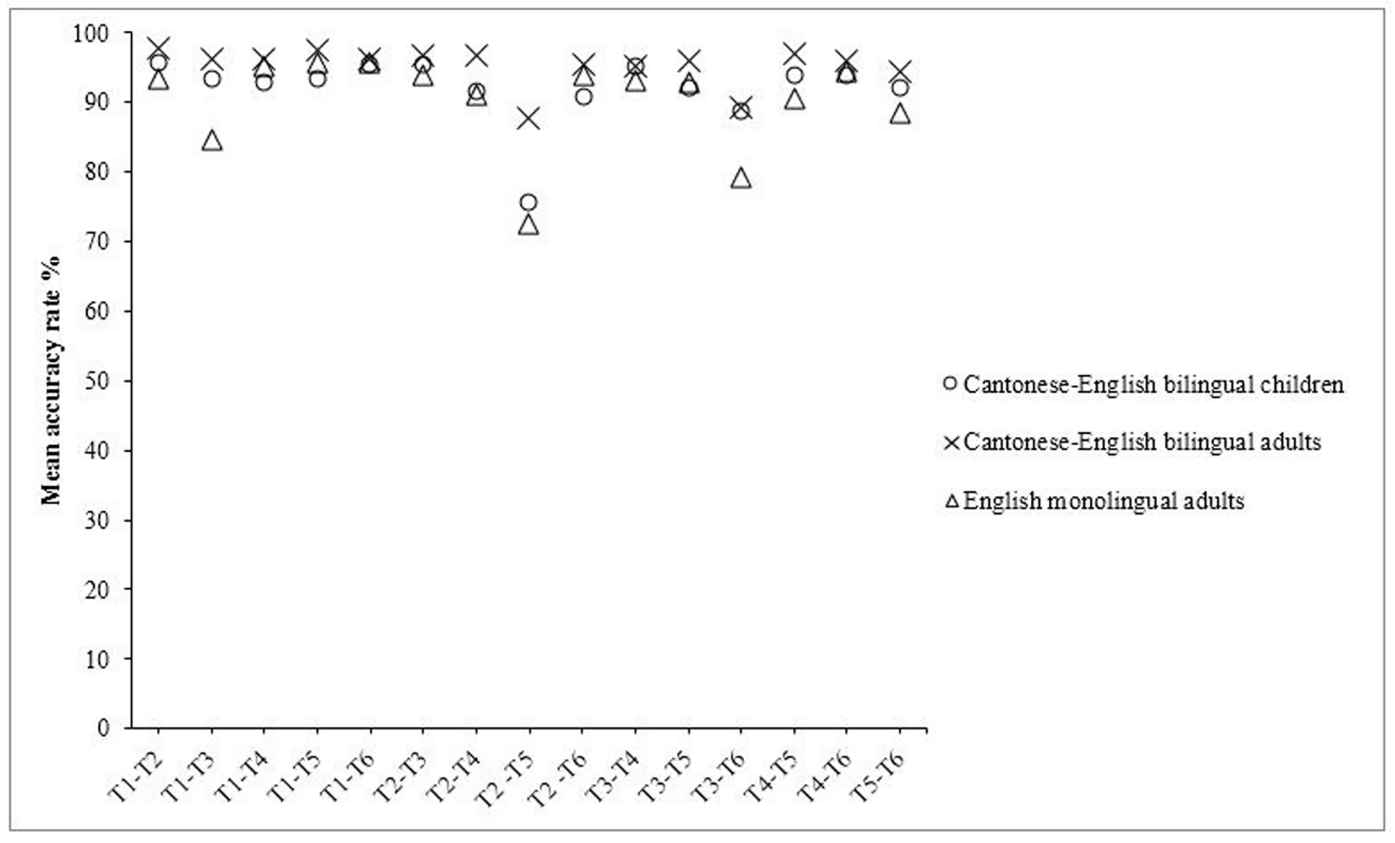
Mean Percentages of correct response for all 15 tone contrasts for all three groups.

Fig 3 shows mean accuracy rates for the 15 Cantonese tone contrasts in each of the three participant groups. To examine if the patterns of tone perception vary across three groups, we first conducted an overall omnibus test on the effects of group and tone contrast by using a 3 × 15 mixed-design analysis of variance (ANOVA) with group as a between-subjects factor and tone contrast as a within-subjects factor. There was a significant main effect of group, *F*(2, 72) = 5.16, *p* < .01, η^2^_p_ = .13, and a significant main effect of tone contrast, *F*(14, 1008) = 24.38, *p* < .001, η^2^_p_ = .25. The crucial result was a significant interaction between group and tone contrast, *F*(28, 1008) = 2.46, *p* < .001, η^2^_p_ = .06. Thus, in the subsequent analyses, we examined group differences in each individual tone contrast. We also examined the perceptual differences of 15 tone contrasts separately for each group.

The simple main effect analysis of group difference in each individual tone contrast showed that the three groups’ accuracy rates differed for the tone contrasts T2-T5 [*F*(2, 72) = 7.61, *p <* .01, η^2^_p_ = .17]; T2-T4 [*F*(2, 72) = 3.76, *p <* .05, η^2^_p_ = .10]; T1-T3 [*F*(2, 72) = 8.28, *p <* .01, η^2^_p_ = .19]. Post-hoc tests with Bonferroni correction revealed that Cantonese-English bilingual adults’ accuracy scores were higher than those of English monolingual adults for all of these tone contrasts (*p*s *<* .05). However, there were no differences between Cantonese-English bilingual children and English monolingual adults on accuracy for these tone contrasts (*p*s > .05), with the T1-T3 contrast being the only exception (*p <* .05). Cantonese-English bilingual adults’ accuracy on the T2-T5 contrast was significantly higher than that of Cantonese-English bilingual children (*p* < .05); however, there were no other significant differences between the bilingual adults and children.

There was a trend that three groups’ accuracy rates differed in T3-T6 [*F*(2, 72) = 2.83, *p* = .07, η^2^_p_ = .07]; T2-T6 [*F*(2, 72) = 2.72, *p* = .07, η^2^_p_ = .07]; T4-T5 [*F*(2, 72) = 2.89, *p* = .06, η^2^_p_ = .07]. Post-hoc tests with Bonferroni correction revealed that Cantonese-English bilingual adults’ accuracy rates tended to be higher than that of English monolingual adults (*p*s *<* .10) only; there were no significant differences in these tone contrasts between Cantonese-English bilingual adults and children (*p*s > .05), as well as Cantonese-English bilingual children and English monolingual adults(*p*s > .05).

We also conducted a separate ANOVA for each group to compare the mean accuracy scores for all 15 tone contrasts. For Cantonese-English bilingual children, there was a significant difference in accuracy rates across the 15 tone contrasts, *F*(14, 406) = 12.03, *p* < .001, η^2^_p_ = .29. Pairwise comparisons with Bonferroni correction revealed that their performance on T2-T5 (*M* = .75) was significantly lower than their performance on all other tone contrasts (*p*s < .05). There were no significant differences in performance between other contrasts (*p*s > .05).

Cantonese-English bilingual adults also showed a significant main effect of tone contrasts, *F*(14, 406) = 6.00, *p* < .001, η^2^_p_ = .17. Pairwise comparisons with Bonferroni correction revealed that their performance on the T2-T5 contrast (*M* = .88) was significantly lower than that of T1-T2 (*p* < .05); T1-T3 (*p* < .05); T1-T5 (*p* < .05); T2-T3 (*p* < .05); T2-T4 (*p* < .05); T4-T5 (*p* < .05); T4-T6 (*p* < .05). Their performance on the T3-T6 contrast (*M* = .89) was also lower than their performance on T1-T2 (*p* < .05); T2-T3 (*p* = .08); T1-T5 (*p* < .05); T3-T5 (*p* < .05); T1-T4 (*p* < .05); and T4-T5 (*p* = .06). There were no significant differences in performance between other contrasts (*p*s > .05).

For English monolingual adults, there were significant differences in accuracy across the 15 tone contrasts, *F*(14, 196) = 7.53, *p* < .001, η^2^_p_ = .35. Pairwise comparisons with Bonferroni correction revealed that their performance on T2-T5 (*M* = .73) was lower than all other contrasts (*p*s < .05) except for the contrasts T4-T5, T2-T4, T5-T6, T1-T3, and T3-T6 (*p*s > .05). Their performance on the T1-T3 contrast (*M* = .85) was also significantly lower than that of T1-T5 (*p* < .05) and was marginally lower than that of T1-T6 (*p* = .08).

To summarize, bilingual children reached adult levels of accuracy on most of the 15 tone contrasts (see Fig 3), but not all—accuracy on the T2-T5 contrast, specifically, was low. Moreover, bilingual children had better performance than English monolingual adults on T1-T3, but this was the only tone contrast on which they outperformed the adults.

### Acoustic Analyses of Cantonese Lexical Tone and English Stress

Our two perception tests each included a large set of tone (660 tokens) and stress (110 tokens) stimuli, which allowed us to conduct a systematic analysis of all possible acoustic correlates of Cantonese tones and English stress patterns to examine the critical acoustic correlates that determine the perceptual saliency of Cantonese tone and English stress. Using Praat acoustic software [45], we extracted a wide range of acoustic parameters: vowel duration (in ms), fundamental frequency (F0, in Hz), average intensity (in dB), spectral balance, and formant frequency (F1 and F2), all of which have been demonstrated in different studies to be used by speakers in perceiving or producing tone or stress [15,16].

In our analyses, there were a total of 660 Cantonese tone tokens and 110 English stress tokens. Each token was measured according to all the above listed acoustic parameters. For Cantonese tone tokens, we analyzed all tone syllables with tone placing on the first syllable given that there was no perceptual difference in perceiving tones in the first or the second syllable. The analyses were based on the acoustic measures of all tokens of the same tone. There were 110 tokens for each tone, whereas there were 55 tokens for each type of stress pattern. The analyses of tone are reported first, followed by analyses of stress.

### Acoustic Correlates of Six Cantonese Tones

Table 1 shows means and standard deviations of duration, average F0, F0 onset, F0 offset, F0 general slope, F0 displacement, F0 major slope, intensity, F1, and F2 for all six Cantonese tones. An ANOVA was carried out to examine differences among the six Cantonese tones in terms of each of those acoustic parameters.

**Table 1:** Means, Standard Deviations, and Comparisons between Different Acoustic Parameters for the Six Cantonese Tones.

	T1		T2		T3		T4		T5		T6		
	*M*	*SD*	*M*	*SD*	*M*	*SD*	*M*	*SD*	*M*	*SD*	*M*	*SD*	*F*(5, 270)
Duration	230.64	30.09	311.62	41.34	250.68	29.45	228.23	29.93	288.05	34.02	229.16	28.52	131.03***
F0 Avg.	303.48	5.04	206.06	9.10	258.13	4.84	184.59	6.01	207.08	5.82	225.79	4.09	3547.93***
F0 Onset	315.99	10.89	217.20	14.33	273.40	9.05	211.79	14.88	220.84	16.43	247.93	12.32	1037.14***
F0 Offset	290.45	7.21	256.73	11.91	254.19	4.79	182.01	7.66	239.90	8.10	224.13	6.90	1315.69***
F0 G. S.	-111.48	55.05	+131.30	70.09	-77.90	41.85	-132.76	81.50	+65.49	60.79	-105.63	67.41	289.14***
F0 Displace.	-25.54	12.50	+39.52	19.13	-19.21	9.95	-29.77	17.31	+19.05	18.13	-23.80	14.50	322.60***
F0 M. S.	-111.48	55.05	+379.72	93.98	-77.90	41.85	-233.21	112.89	+286.24	49.58	-105.63	67.41	745.50***
Intensity	65.71	1.78	62.72	1.74	65.12	1.35	62.61	2.62	64.17	2.11	62.42	1.61	70.30***
F1	569.08	159.98	571.68	208.77	561.02	169.05	550.85	198.79	573.60	203.30	569.41	166.60	1.95
F2	1765.64	585.12	1785.53	763.84	1805.49	676.62	1746.36	785.32	1793.22	690.80	1763.43	667.90	1.17

*Note*.–indicates a falling direction while + indicates a rising direction. F0 Avg = F0 average. F0 G. S. = F0 general slope. F0 Displace = F0 displacement. F0 M. S. = F0 major slope.

***p < .001.

#### Duration

For our purposes, duration refers specifically to vowel duration in milliseconds. Results of the ANOVA of average duration across the six tones showed a significant main effect of tone category, *F*(5, 270) = 131.03, *p* < .001, η^2^_p_ = .71. Pairwise comparisons with Bonferroni correction suggested that T1, T4, and T6 duration values were statistically indistinguishable from one another (*p*s = 1.00). By contrast, the duration values for T2, T5, and T3 were statistically different from one another (*p*s < .001), with high rising T2 having the longest duration (*M* = 311.62ms) followed by the low rising tone T5 (*M* = 288.05ms) and then by the level tone T3 (*M* = 250.68ms). Also, their duration values were all significantly greater than that of T1, T4, and T6 (*p*s < .001).

#### Tone Height

There were three different measures of tone height: average F0, F0 onset and F0 offset. For the average F0, there was a significant main effect of tone category, *F*(5, 270) = 3547.93, *p* < .001, η^2^_p_ = .99. Pairwise comparisons with Bonferroni correction revealed that the average F0s of T2 (*M* = 206.06Hz) and T5 (*M* = 207.08Hz) were statistically indistinguishable (*p* = 1.00), but the average F0 values of all other tones were significantly different from one another (*p*s < .001). As shown in Table 1, T1 had the highest average F0 (*M* = 303.48Hz), followed by T3 (*M* = 258.13Hz) and T6 (*M* = 225.79Hz). T4 had the lowest average F0 value (*M* = 184.59Hz).

With regard to F0 onset, the main effect of tone category was significant, *F*(5, 270) = 1037.14, *p* < .001, η^2^_p_ = .95. Pairwise comparisons with Bonferroni correction suggested that T2’s F0 onset (*M* = 217.20Hz) was statistically indistinguishable from that of T5 (*M* = 220.84 Hz, *p* = 1.00) and T4 (*M* = 211.79, *p* = .09). But the mean F0 onset values of all other tones were significantly different from one another (*p*s < .001), with T1 having the highest F0 onset value (*M* = 315.99Hz), followed by T3 (*M* = 273.40Hz) and T6 (*M* = 247.93Hz).

With regard to F0 offset, there was a significant main effect of tone category, *F*(5, 270) = 1315.69, *p* < .001, η^2^_p_ = .96. Pairwise comparisons with Bonferroni correction suggested that T2’s F0 offset (*M* = 256.73Hz) was statistically indistinguishable from that of T3 (*M* = 254.19Hz, *p* = 1.00), but the F0 offset values of all other tones were significantly different from one another (*p*s < .001), with T1 having the highest F0 offset value (*M* = 290.45Hz) followed by T5 (*M* = 239.90Hz), T6 (*M* = 224.13Hz), and T4 (*M* = 182.01Hz).

#### Tone Contour

We first used one simple measure to quantify tone contour: F0 general slope. F0 general slope was defined as the general gradient between F0 onset and F0 offset. It was calculated by dividing the difference between F0 offset and F0 onset by duration. F0 general slope is a directional measure that shows the F0 movement of tones. It was expected that the level tones (T1, T3, T6) would have similar general slopes while rising tones (T2, T5) and falling tone (T4) would have more positive and more negative general slopes, respectively. Also, we expected that the high rising tone (T2) might have a greater positive general slope than the low rising tone (T5).

For F0 general slope, an ANOVA showed that there was a significant main effect of tone category, *F*(5, 270) = 289.14, *p* < .001, η^2^_p_ = .84. Pairwise comparisons with Bonferroni correction suggested that T1 was statistically indistinguishable from T6 (*p* = 1.00) and T4 (*p* = .80); the F0 general slope was -111.48Hz/ms for T1, -105.63Hz/ms for T6, and -132.76Hz/ms for T4. All other pairwise comparisons were statistically significant (*p*s < .05). As shown in Table 1, T2 (+131.30Hz/ms) had the most positive general slope, followed by T5 (+65.49Hz/ms); T3 (-77.90Hz/ms) had a shallower negative slope than T6, T1, and T4.

We noticed that T3, a mid-level tone, had a significantly different F0 general slope than the two other level tones, T1 and T6. Since T3 was shown to have longer duration (*M* = 250.68ms) than both T1 (*M* = 230.64ms) and T6 (*M* = 229.16ms), the question arose as to whether such duration differences led to the difference in F0 general slope between the three level tones. Thus, we took out the time factor from our F0 general slope measure and examined F0 displacement. F0 displacement was a directional measure of a change from F0 onset to F0 offset.

Results of the analysis of F0 displacement showed that there was a significant main effect of tone category, *F*(5, 270) = 322.60, *p* < .001, η^2^_p_ = .86. Pairwise comparisons with Bonferroni correction suggested that T3 (*M* = -19.21Hz) was statistically indistinguishable from T6 (*M* = -23.80Hz), *p* = .08, while T1 (*M* = -25.54Hz) was statistically indistinguishable from T4 (*M* = -29.77Hz), *p* = 1.00, and T6 (*p* = 1.00). But the F0 displacement values of all other tones were statistically significant (all *p*s < .05), with T2 (*M* = +39.52Hz) having the greatest positive change in F0 followed by T5 (*M* = +19.05Hz). These results showed that, without taking into account the duration, the level tones and the falling tone had similar overall tone contour.

The results presented up until now showed that high level T1 and low falling T4 had similar F0 general slopes and F0 displacements, which indicated similar tone contour. We speculated that these two measures of tone contour, i.e., both F0 general slope and F0 displacement, were influenced by tonal context effects resulting from the disyllabic pseudowords used in the present study. In those disyllabic stimuli, the first syllable carried the experimental tones ranging from tone 1 to 6, whereas the second syllable always carried the high level tone T1 (a control tone). Since T4 had the lowest F0 offset (182.01Hz) among all the tones, its F0 offset would be substantially affected by the following T1, which has the highest F0 onset among all other tones (315.99 Hz). This design feature may lead to a tonal context effect in the F0 offset of T4, thereby making the F0 general slope value and F0 displacement of T4 less negative than it might be otherwise.

Indications of such a tonal context effect are evident in Fig 1A. According to the F0 traces depicted in Fig 1A, the contours of the three level tones, i.e., high level T1, mid level T3, and low level T6, were linear. But the contour of T4 was a concave upward curve: the F0 movement tendency of T4 gradually changes from falling to rising when it comes closer to its end. This could be due to the influence of the following high level T1. Similarly, the two rising tones T2 and T5 also exhibited an obvious concave upward curve. On the basis of our results, it seems that the F0 general slope measure was not sensitive to a concave function for tone contours. Therefore, we conducted a fine-grained analysis of the tone contours of the rising tones and falling tone by examining the falling and rising portions of the F0 traces for T2, T4, and T5.

#### Falling Contour for T2, T4, and T5

The falling portions of T2, T4, and T5’s contours were examined using three different acoustic parameters: F0 fall displacement, F0 fall time, and F0 fall slope. F0 fall displacement was defined as a change in F0 from F0 onset to F0 minimum (in Hz). F0 fall time was the duration between F0 onset and F0 minimum (in ms). F0 fall slope referred to the gradient between F0 minimum and F0 onset (in Hz/ms). It was calculated by dividing F0 fall displacement by F0 fall time. Table 2 displays the means and standard deviations of F0 fall displacement, F0 fall time, and F0 fall slope for T2, T4, and T5.

**Table 2:** Means, Standard Deviations, and Comparisons between Contour Acoustic Parameters for T2, T4 and T5.

	T2		T4		T5		
	M	SD	M	SD	M	SD	*F*(2, 108)
F0 Fall Displace.	-36.50	13.29	-36.30	15.71	-33.75	15.28	1.47
F0 Fall Time	108.34	25.74	166.86	49.32	103.61	22.16	55.05***
F0 Fall Slope	-351.37	135.86	-233.21	112.89	-337.93	168.73	26.59***
F0 Rise Displace.	+75.40	14.16	+6.53	7.09	+52.20	9.30	666.62***
F0 Rise Time	205.01	39.52	61.37	40.76	184.43	30.00	256.03***
F0 Rise Slope	+379.72	93.98	+84.80	87.02	+286.24	49.58	219.58***
R-F D-ratio	2.40	1.37	.24	.31	2.14	1.63	64.26***
R-F T-ratio	2.01	.65	.46	.43	1.90	.68	119.37***

*Note*.–indicates a falling direction while + indicates a rising direction. F0 Fall Displace. = F0 fall displacement. F0 Rise Displace. = F0 rise displacement.

***p < .001.

Separate ANOVAs were conducted to examine differences between the three Cantonese tones (T2, T4, and T5) in terms of F0 fall displacement, F0 fall time, and F0 fall slope separately. Results of the analysis of F0 fall displacement showed that there was no significant main effect of tone category, *F*(2, 108) = 1.47, *p* = .24, with T2 (*M* = -36.50Hz), T4 (*M* = 36.30Hz), and T5 (*M* = -33.75Hz) having similar F0 fall displacements.

By contrast, the analysis of F0 fall time showed a significant main effect of tone category, *F*(2, 108) = 55.05, *p* < .001, η^2^_p_ = .51. Pairwise comparisons with Bonferroni correction showed that only T2’s F0 fall time (*M* = 108.34ms) and T5’s F0 fall time (*M* = 103.61ms) were statistically indistinguishable from each other (*p* = .73). But T4 (*M* = 166.86ms) had a longer F0 fall time than both T2 (*p* < .001) and T5 (*p* < .001).

Similarly, there was a significant main effect of tone category in F0 fall slope, *F*(2, 108) = 26.59, *p* < .001, η^2^_p_ = .33. Pairwise comparisons with Bonferroni correction suggested that T2’s F0 fall slope (*M* = -351.37Hz/ms,) was statistically indistinguishable from T5 (*M* = -337.93Hz/ms). T4 had a less negative fall slope (*M* = -233.21Hz/ms) than both T2 (*p* < .001) and T5 (*p* < .001).

Up to this point, our results indicate that T2 and T5 shared similar falling portions, and that they were both different from T4. As shown in our earlier analyses, T2 and T5 also had similar F0 onset, F0 fall displacement, F0 fall time, and F0 fall slope. Thus, we further analyzed the rising contours of these three contour tones in order to examine whether rising contour was the key determiner of the perceptual distinction between T2 and T5.

#### Rising Contour for T2, T4 and T5

We used three similar measures to examine the rising portions of the contours of T2, T4, and T5: F0 rise displacement, F0 rise time, and F0 rise slope. F0 rise displacement referred to a change in F0 from F0 minimum to F0 offset (in Hz). F0 rise time referred to the duration between F0 minimum and F0 offset (in ms). F0 rise slope referred to the gradient between the F0 offset and F0 minimum (in Hz/ms) and it was calculated by dividing F0 rise displacement by F0 rise time. Table 2 shows means and standard deviations of F0 rise displacement, F0 rise time, and F0 rise slope for T2, T4, and T5.

Results of the analysis of F0 rise displacement showed a significant main effect of tone category, *F*(2, 108) = 666.62, *p* < .001, η^2^_p_ = .93, with the F0 rising displacement values of all three tones—i.e., T2 (*M* = +75.40, Hz), T5 (*M* = +52.20Hz), and T4 (*M* = +6.53Hz)—being significantly different from one another (*p*s < .001). Similar results were also obtained for both F0 rise time, *F*(2, 108) = 256.03, *p* < .001, η^2^_p_ = .83, and F0 rise slope, *F*(2, 108) = 219.58, *p* < .001, η^2^_p_ = .80. Specifically, the F0 rise time of T2 (*M* = 205.01ms), T5 (*M* = 184.43ms), and T4 (*M* = 61.37ms), (ranked in descending order) significantly differed from one another (*p*s ≤ .01). The F0 rise slope values of T2 (*M* = +379.72Hz/ms), T5 (*M* = +286.24Hz/ms), and T4 (*M* = +84.80Hz/ms) also significantly differed from one another (*p*s ≤ .001). Thus, T2, T4, and T5 had distinctive rising portions.

Further, we calculated the ratio of F0 rise displacement to F0 fall displacement, referred to as the R-F D-ratio, and the ratio of F0 rise time to F0 fall time, referred to as the R-F T-ratio, in order to directly compare the rising portions to the falling portions of T2, T4, and T5’s contours. Table 2 shows means and standard deviations of the R-F D-ratios and R-F T-ratios for T2, T4, and T5. Results of an ANOVA on the R-F D-ratio showed a significant main effect of tone category, *F*(2, 108) = 64.26, *p* < .001, η^2^_p_ = .54. Pairwise comparisons with Bonferroni correction suggested that there was no difference between T2 (*M* = 2.40) and T5 (*M* = 2.14) in R-F D-ratio (*p* = .76), but each of them was significantly larger than T4 (*M* = .24) in R-F D-ratio (*p*s < .001). Similarly, the main effect of tone category was also significant in an analysis of the R-F T-ratio, *F*(2, 108) = 119.37, *p* < .001, η^2^_p_ = .69. Pairwise comparisons with Bonferroni correction suggested that the two rising tones T2 (*M* = 2.01) and T5 (*M* = 1.90) were statistically indistinguishable from each other (*p* = .96), and that each of them was significantly larger than T4 (*p* < .05).

To summarize, our analyses of the falling and rising portions of tones’ contours showed that T2 and T5 were significantly different from each other in the rising portions but not in the falling portions, and that the length of the rising portions of T2 and T5 were about twice the length of their own falling portion. These results raised the possibility that a listener in the present study might just focus on the rising portion in their perceptual discrimination of T2 and T5. Conversely, for T4, a listener might just focus on the falling portion in perceiving the contextually-affected T4 tone in this study.

To examine this possibility, we used another F0 slope measure, F0 major slope, to compare the most distinctive tone contour among the six Cantonese lexical tones. The F0 major slope referred to the F0 rising slope for the rising tones T2 and T5, while the F0 major slope for the falling tone T4 was its falling slope. For the level tones T1, T3, and T6, their F0 major slopes were the same as their F0 general slopes.

Results of the ANOVA of the F0 major slope showed that there was a significant main effect of tone category, *F*(5, 270) = 745.50, *p* < .001, η^2^_p_ = .93. Pairwise comparisons with Bonferroni correction suggested that the F0 major slope values of T1 and T6 were statistically indistinguishable from each other (*p* = 1.00), and that all other pairwise comparisons were statistically significant (*p*s < .01). The tones’ F0 major slopes, presented in descending order from the most positive to the most negative, were: T2 (*M* = +379.72Hz/ms), T5 (*M* = +286.24Hz/ms), T3 (*M* = -77.90Hz/ms), T6 (*M* = -105.63Hz/ms), T1 (*M* = -111.48Hz/ms), and T4 (*M* = -233.21Hz/ms). Thus, F0 major slope could serve as the quantifier for categorizing Cantonese lexical tones into rising, level, and falling tones: the slope range of +270 to +410 was rising tones, -70 to -130 was for level tones, and -200 to -260 was for falling tone (using 95% confidence intervals).

#### Intensity

An ANOVA of average intensity (measured in dB) showed a significant main effect of tone category, *F*(5, 270) = 70.30, *p* < .001, η^2^_p_ = .57. Pairwise comparisons with Bonferroni correction suggested that T2 (*M* = 62.72dB), T4 (*M* = 62.61dB), and T6 (*M* = 62.42dB) were statistically indistinguishable from one another (*p*s = 1.00), and that all other pairwise comparisons were statistically significant (*p*s < .05), with T1 (*M* = 65.71dB) having the highest intensity followed by T3 (*M* = 65.12dB) and T5 (*M* = 64.17dB).

#### Formant Frequencies Measures

Separate ANOVAs on the formant frequencies F1 and F2 showed no significant main effects of tone category; *F*(5, 270) = 1.95, *p* > .05 for F1, and *F*(5, 270) = 1.17, *p* > .05 for F2, respectively. These results indicate that the production of Cantonese lexical pitch and its carrying vowels are independent of each other.

#### Spectral Balance

Consistent with Sluijter and van Heuven’s (1996) study of spectral balance on English lexical stress, in this study spectral balance was measured in four contiguous frequency bands: Band 1 (0-500Hz), Band 2 (500-1000Hz), Band 3 (1000-2000Hz), and Band 4 (2000-4000Hz). Spectral balance was investigated only on the vowels of the CVCV stimuli. Band energy of each frequency band for a vowel was obtained from Praat by analyzing its stable portion of no less than 70ms in duration. To remove the factor of duration in the analysis of spectral balance, band energy in squared pressure seconds (Pa^2^sec) was converted to sound pressure level (dB) by the following formula:

$$Sound\;pressure\;level\;\text{in dB}=10{\text{log}}_{10}\left(\frac{\frac{Band\;energy\;\text{in Pa}2\;\text{sec}}{time\;\text{in second}}}{4\times 10^{-10}\;\text{in Pa}2\;\text{sec}}\right)$$

Table 3 shows the means and standard deviations of B1, B2, B3, and B4 for the six Cantonese tones. To examine differences in the spectral balance of the tones, we carried out a two-way repeated measures ANOVA with both tones (T1, T2, T3, T4, T5, T6) and band category (B1, B2, B3, B4) as within-subject factors. The analysis showed a significant main effect of tone category, *F*(5, 270) = 125.21, *p* < .001, η^2^_p_ = .70. Pairwise comparisons with Bonferroni correction suggested that the overall average spectral energy of T1 (*M* = 49.49, *SD* = 10.39) and T3 (*M* = 49.23, *SD* = 10.69) were statistically indistinguishable from each other (*p* = 1.00), and that of T5 (*M* = 45.89, *SD* = 12.16) and T6 (*M* = 45.42, *SD* = 11.29) were also statistically indistinguishable from each other (*p* = 1.00). However, all other pairwise comparisons of overall spectral energy were statistically significant (*p*s < .05). The main effect of band category was also significant, *F*(3, 162) = 69.73, *p* < .001, η^2^_p_ = .56. Pairwise comparisons with Bonferroni correction suggested that only B3 (*M* = 38.12, *SD* = 12.82) and B4 (*M* = 38.44, *SD* = 9.88) were statistically indistinguishable from each other (*p* = 1.00), and that the band energy of both B3 and B4 was significantly lower than that of B1 (*M* = 55.41, *SD* = 3.10; *p*s < .001) and B2 (*M* = 51.88, *SD* = 8.45; *p*s < .001), with B1 having the greatest band energy. The interaction effect between tone category and band category was also significant, *F*(15, 810) = 22.87, *p* < .001, η^2^_p_ = .30.

**Table 3:** Means and Standard Deviations of B1, B2, B3 and B4 for the Six Cantonese Tones.

	B1		B2		B3		B4	
	M	SD	M	SD	M	SD	M	SD
T1	57.03	2.35	56.11	6.31	42.78	11.07	42.05	8.05
T2	53.51	3.38	48.96	8.48	35.21	12.85	36.61	9.57
T3	57.46	1.42	55.99	3.74	42.94	11.37	40.51	9.20
T4	53.51	3.44	50.34	8.78	30.67	13.19	34.12	11.08
T5	55.05	3.31	52.16	8.91	37.64	12.62	38.69	10.11
T6	55.88	1.50	47.70	9.39	39.46	11.76	38.65	9.43

A follow-up simple main effect analysis indicated that all six tones have different band energy in Band 1, *F*(5, 270) = 60.12, *p* < .001, η^2^_p_ = .53. Pairwise comparisons with Bonferroni correction suggested that three pairs of tones, namely T1 and T3 (*p* = 1.00), T2 and T4 (*p* = 1.00), and T5 and T6 (*p* = .39), were statistically indistinguishable from one another, while all other pairwise comparisons of overall spectral energy were statistically significant (*p*s < .05).

For Band 2, the main effect of tone category was significant, *F*(5, 270) = 51.32, *p* < .001, η^2^_p_ = .49. Pairwise comparisons with Bonferroni correction suggested that three pairs of tones were statistically indistinguishable from one another: T1 and T3 (*p* = 1.00), T2 and T4 (*p* = .16), T2 and T6 (*p* = 1.00); all other pairwise comparisons of overall spectral energy were statistically significant (*p*s < .05).

For Band 3, the main effect of tone category was significant, *F*(5, 270) = 94.95, *p* < .001, η^2^_p_ = .64. Pairwise comparisons with Bonferroni correction suggested that only two pairs of tones, T1 and T3 (*p* = 1.00) and T5 and T6 (*p* = .06), were statistically indistinguishable from each other, while all other pairwise comparisons of overall spectral energy were statistically significant (*p*s < .05).

**Fig 4:**
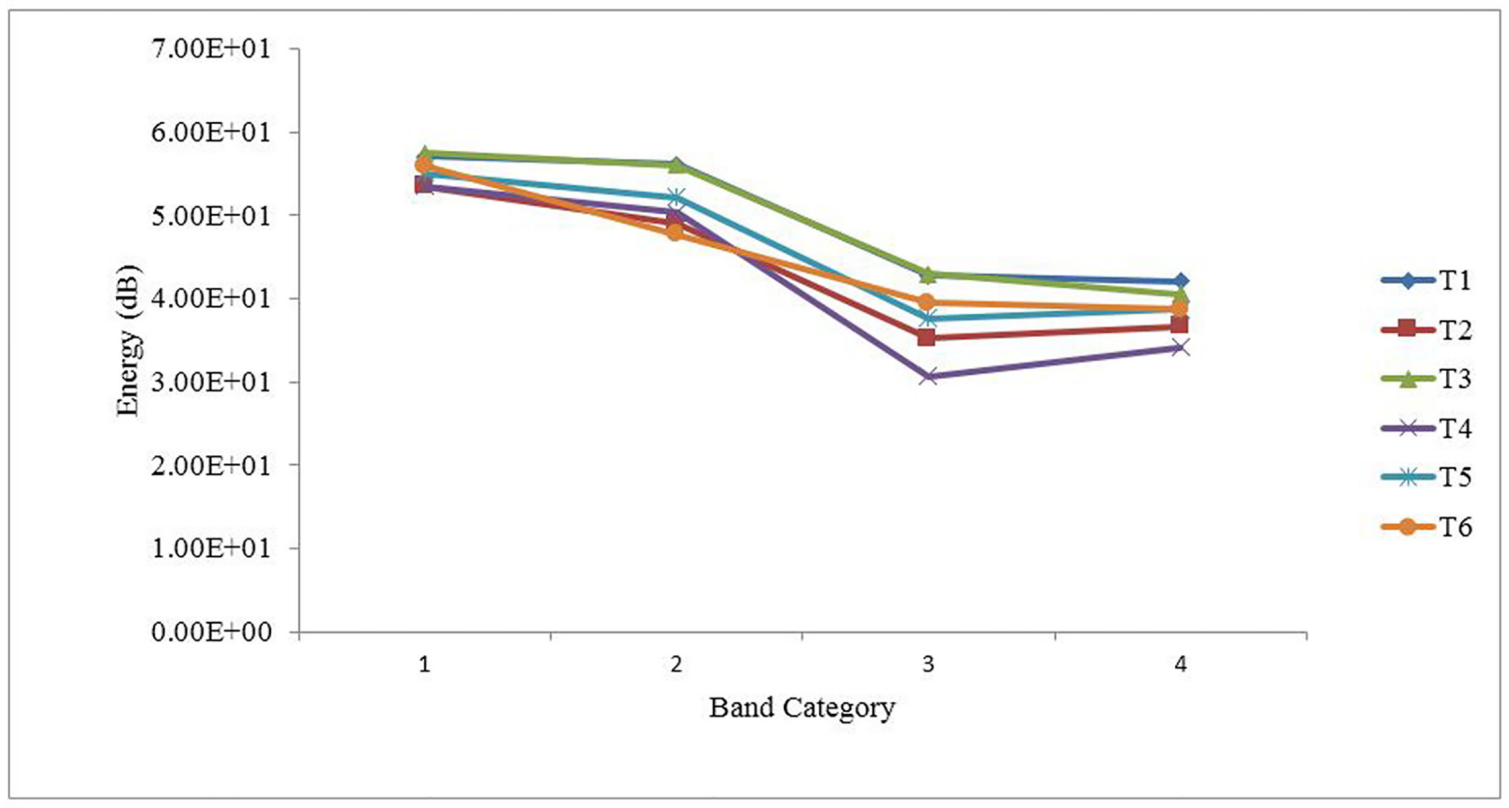
Spectral energy of the six Cantonese lexical tones across the four bands.

For Band 4, the main effect of tone category was significant, *F*(5, 270) = 35.84, *p* < .001, η^2^_p_ = .40. Pairwise comparisons with Bonferroni correction suggested that three pairs of tones, T1 and T3 (*p* = 1.00), T2 and T6 (*p* = .12), T5 and T6 (*p* = 1.00), were statistically indistinguishable from one another, while all other pairwise comparisons of overall spectral energy were statistically significant (*p*s < .05). Thus, only T1 and T3 shared the same spectral energy across the four bands, as depicted in Fig 4.

#### Estimated Importance of Different Acoustic Correlates of Cantonese Tone

To further estimate the overall percentage variation of attributable to different acoustic correlates of Cantonese tone, we divided the sum of the absolute differences between the mean of the acoustic correlates of all 15 tone contrasts by the sum of the absolute mean of the acoustic correlates of all six Cantonese tones. The equation is shown below. Using that equation, the estimated overall variation in all tone contrasts accounted for by the different acoustic correlates, in descending order, were: average F0 (56%), F0 onset (48%), F0 offset (45%), duration (40%), F0 major slope (38%), B3 (38%), B4 (22%), B2 (21%), B1 (9%), Intensity (6%).

$$Estimated\;overall\;variation=\frac{{\left|{\bar{X}}_{T2}-{\bar{X}}_{T5}\right|}_{1}+{\left|{\bar{X}}_{T3}-{\bar{X}}_{T6}\right|}_{2}+\cdots +{\left|{\bar{X}}_{T1}-{\bar{X}}_{T2}\right|}_{15}}{\left|{\bar{X}}_{T1}|+|{\bar{X}}_{T2}|+|{\bar{X}}_{T3}|+|{\bar{X}}_{T4}|+|{\bar{X}}_{T5}|+|{\bar{X}}_{T6}\right|}$$

Of all 10 possible acoustic correlates of Cantonese tones, then, intensity had the lowest estimated overall variation. Since the intensity cue is very subtle compared to other acoustic cues in terms of estimated overall variation, a native Cantonese speaker may attend to other more salient and more reliable cues. Also, intensity is a weak cue for stress perception, suggesting that it may not be useful to native English speakers. Thus, the intensity cue was not significantly used by any of the three groups in this study.

In addition, it is interesting that although the tone contour measure of F0 major slope had very large estimated overall variation, and although 14 out of 15 tone contrasts (93%) had statistically different F0 major slope, only adult bilinguals could use tone contour as one of the general perceptual mechanisms to process tones across all tone contrasts.

### English Lexical Stress

Although there were only two types of lexical stress in our disyllabic stimuli, there could be four different lexical stress conditions (stress × syllable position) namely: (1) the first syllable of trochaic (strong-weak) stressed stimuli (**TS1**), e.g. /**'la**mu/; (2) the second syllable of trochaic (strong-weak) stressed stimuli (**TS2**), e.g. /'la**mu**/; (3) the first syllable of iambic (weak-strong) stressed stimuli (**IS1**), e.g. /**la**'mu/; and (4) the second syllable of iambic (weak-strong) stressed stimuli (**IS2**), e.g. /la**'mu**/. Table 4 shows the means and standard deviations of duration, average F0, F0 onset, F0 offset, F0 general slope, intensity, F1 and F2 for these four conditions. Separate one-way ANOVAs were conducted for each of these acoustic parameters to examine differences of the four stress conditions.

**Table 4:** Means, Standard Deviations, and Comparisons between Different Acoustic Parameters for the Four Stress Conditions.

	TS1		TS2		IS1		IS2		
	M	SD	M	SD	M	SD	M	SD	*F*(3, 162)
Duration	228.34	28.27	168.21	34.05	207.99	29.31	280.57	28.74	177.04***
F0 Avg.	317.96	44.55	187.25	9.93	232.90	5.95	245.53	11.87	293.56***
F0 Onset	329.45	13.50	239.84	17.53	257.49	12.69	287.10	15.30	434.10***
F0 Offset	309.69	14.02	149.87	16.13	222.81	7.03	182.15	16.30	1394.33***
F0 G. S.	-86.43	70.82	-548.96	137.75	-169.37	62.56	-377.66	76.67	306.97***
Intensity	66.60	1.53	59.69	2.13	62.57	2.06	64.12	1.73	169.10***
F1	565.26	148.89	595.37	196.88	592.92	156.23	621.63	176.41	1.32
F2	1788.43	651.09	1857.28	685.45	1803.08	677.50	1848.60	630.67	.172

*Note*. F0 Avg. = F0 average. F0 G. S. = F0 general slope.

***p < .001.

#### Duration

As in the Cantonese tone analysis described above, duration refers specifically to vowel duration. The ANOVA showed that there was a significant main effect of stress condition, *F*(3, 162) = 177.04, *p* < .001, η^2^_p_ = .77. Pairwise comparisons with Bonferroni correction suggested that the duration values of all stress conditions were statistically different from one another (*p*s < .001). The duration values of these four conditions, in descending order, were: IS2 (*M* = 280.57ms), TS1 (*M* = 228.34ms), IS1 (*M* = 207.99ms) and TS2 (*M* = 168.21ms).

#### Pitch Height

We analyzed three pitch height measures: average F0, F0 onset and F0 offset. With regard to the average F0, a one-way ANOVA showed a significant main effect of stress condition, *F*(3, 162) = 293.56, *p* < .001, η^2^_p_ = .85. Pairwise comparisons with Bonferroni correction suggested that the average F0 values of all stress conditions were statistically different from one another (*p*s < .001). The average F0 values for these four stress conditions, in descending order, were: TS1 (*M* = 317.96Hz), IS2 (*M* = 245.53Hz), IS1 (*M* = 232.90Hz) and TS2 (*M* = 187.25Hz).

For F0 onset, the ANOVA showed that there was a significant main effect of stress condition, *F*(3, 162) = 434.10, *p* < .001, η^2^_p_ = .89. Pairwise comparisons with Bonferroni correction suggested that the F0 onset values of all stress conditions were statistically different from one another (*p*s < .001). The F0 onset values for these four stress conditions, in descending order, were: TS1 (*M* = 329.45Hz), IS2 (*M* = 287.10Hz), IS1 (*M* = 257.49Hz) and TS2 (*M* = 239.84Hz).

For F0 offset, the ANOVA showed a significant main effect of stress condition, *F*(3, 162) = 1394.33, *p* < .001, η^2^_p_ = .96. Pairwise comparisons with Bonferroni correction suggested that the F0 offset values of all stress conditions were statistically different from each other (*p*s < .001). The F0 offset values for the four stress conditions, in descending order, were: TS1 (*M* = 309.69Hz), IS1 (*M* = 222.81Hz), IS2 (*M* = 182.15Hz) and TS2 (*M* = 149.87Hz).

#### Pitch Contour

Results of the ANOVA on F0 general slope showed a significant main effect of stress condition, *F*(3, 162) = 306.97, *p* < .001, η^2^_p_ = .85. Pairwise comparisons with Bonferroni correction suggested that the F0 general slopes of all stress conditions were statistically different from one another (*p*s < .001). The F0 general slope for these four stress conditions, ranked in descending order from least negative to most negative, were TS1 (*M* = -86.43, *SD* = 70.82), IS1 (*M* = -169.37, *SD* = 62.56), IS2 (*M* = -377.66, *SD* = 76.67) and TS2 (*M* = -548.96, *SD* = 137.75).

#### Intensity

Results of the ANOVA on intensity showed a significant main effect of stress condition, *F*(3, 162) = 169.10, *p* < .001, η^2^_p_ = .76. Pairwise comparisons with Bonferroni correction suggested that the intensity values of all stress conditions were statistically different from one another (*p*s < .001). The intensity values for these four stress conditions, in descending order, were: TS1 (*M* = 66.60dB), IS2 (*M* = 64.12dB), IS1 (*M* = 62.57dB) and TS2 (*M* = 59.69dB).

#### Formant Frequencies

Results of the separate ANOVAs on F1 and F2 formant frequencies showed that there were no significant main effects of stress condition in either F1, *F*(3, 162) = 1.32, *p* = .27, or F2, *F*(3, 162) = .172, *p* = .92.

#### Spectral Balance

**Fig 5:**
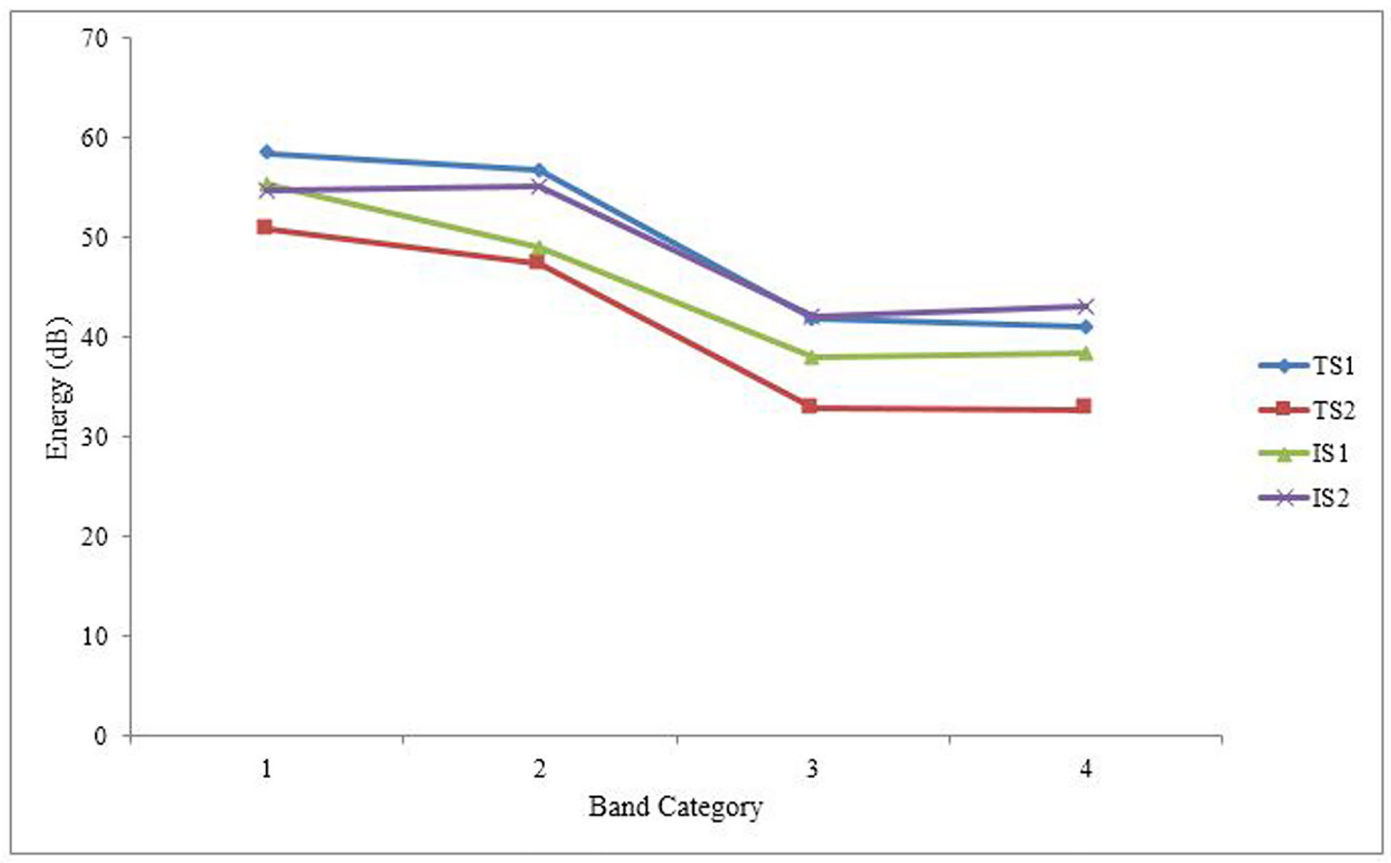
Spectral energy of the four stress conditions across the four bands.

Fig 5 shows spectral balance of TS1, TS2, IS1 and IS2 for English lexical stress. To examine differences in spectral balance, a two-way repeated measures ANOVA was conducted with stress condition (TS1, TS2, IS1 and IS2) and band category (B1, B2, B3, and B4) as within-subject factors. There was a significant main effect of stress condition, *F*(3, 162) = 49.73, *p* < .001, η^2^_p_ = .48. Pairwise comparisons with Bonferroni correction suggested that the overall spectral energy of the two stressed conditions TS1 (*M* = 49.48, *SD* = 11.40) and IS2 (*M* = 48.71, *SD* = 10.03) were statistically indistinguishable from each other (*p* = 1.00), and that all other pairwise comparisons of overall spectral energy were statistically significant (*p*s < .05).

The main effect of band category was also significant, *F*(3, 162) = 143.55, *p* < .001, η^2^_p_ = .73. Pairwise comparisons with Bonferroni correction suggested that B3 (*M* = 38.69, *SD* = 12.85) and B4 (*M* = 38.79, *SD* = 9.51) were statistically indistinguishable from each other (*p* = 1.00), and that the band energy of both B3 and B4 was significantly lower than B1 (*M* = 54.77, *SD* = 4.31; *p*s < .001) and B2 (*M* = 52.02, *SD* = 8.99; *p*s < .001), with B1 having the greatest band energy.

The interaction between stress category and band category was also significant, *F*(9, 486) = 2.54, *p* < .01, η^2^_p_ = .05. To unpack the interaction, we performed simple main effect analyses of stress conditions in each of the different bands. A series of one-way repeated measures ANOVAs with stress conditions as a between-subject factor were conducted. For Band 1, the main effect of stress condition was significant, *F*(3, 162) = 57.48, *p* < .001, η^2^_p_ = .52. Pairwise comparisons with Bonferroni correction suggested that the band energy of IS1 (*M* = 55.25) and IS2 (*M* = 54.71) were statistically indistinguishable from each other (*p* = 1.00), while the other stress conditions were significantly different from one another (*p*s < .001).

For Band 2, there was a significant main effect of stress condition, *F*(3, 162) = 24.72, *p* < .001, η^2^_p_ = .31. Pairwise comparisons with Bonferroni correction suggested that the band energy of TS1 (*M* = 56.70) were statistically indistinguishable from IS2 (*M* = 55.07), and that TS2 (*M* = 47.41) and IS1 (*M* = 48.91) were not different from each other. However, the other comparisons were statistically significant (*p*s < .01).

For Band 3, there was a significant main effect of stress condition, *F*(3, 162) = 8.40, *p* < .001, η^2^_p_ = .14. Pairwise comparisons with Bonferroni correction suggested that the band energy of TS1 (*M* = 41.85) was statistically indistinguishable from IS2 (*M* = 42.00), and that TS2 (*M* = 32.87) and IS1 (*M* = 38.04) were not different from each other. The band energy of ISI (*M* = 38.04) and IS2 (*M* = 42.00) were also statistically indistinguishable. But the band energy of TS1 was significantly greater than TS2 (*p* < .01) and ISI (*p* < .001). Also, the band energy of TS2 was significantly lower than IS2 (*p* < .001).

For Band 4, there was a significant main effect of stress condition, *F*(3, 162) = 17.41, *p* < .001, η^2^_p_ = .24. Pairwise comparisons with Bonferroni correction suggested that the band energy of TS1 (*M* = 40.95) was statistically indistinguishable from IS2 (*M* = 43.04). However, all other comparisons were statistically significant (*p*s < .05), and the comparison between IS1 (*M* = 38.44) and IS2 (*M* = 43.04) was marginally significant (*p* = .09). Thus, only the two stressed conditions, TS1 and IS2, shared the same spectral energy across three band categories (B2, B3 and B4).

### Association between Tone Perception Accuracy and Acoustic Correlates

We further examined the relationship between perceptual performance and acoustic parameters by conducting correlational analyses. Table 5 shows the correlations between the tone perception accuracy of all three groups and all acoustic parameters including duration, average F0, F0 onset, F0 offset, F0 general slope, F0 major slope, intensity, F1, F2, B1, B2, B3, and B4. It is evident that adult bilinguals’ perceptual accuracy was significantly correlated with both child bilinguals’ accuracy (*r* = .86) and adult English monolinguals’ accuracy (*r* = .87), and child bilinguals’ accuracy was also strongly correlated with English monolinguals’ accuracy (*r* = .84), suggesting sufficient shared variance in tone perception accuracy among three groups.

**Table 5:** Correlations between Perceptual Performance and Various Acoustic Correlates of Lexical Tone for Child and Adult Cantonese-English Bilinguals and Adult English Monolinguals.

	1	2	3	4	5	6	7	8	9	10	11	12	13	14	15	16
1. AB P. A.	-															
2. CB P. A.	.86***	-														
3. EM P. A.	.87***	.84***	-													
4. Duration	.29	.10	.13	-												
5. F0 Avg.	.51*	.56*	.62**	-.23	-											
6. F0 Onset	.49*	.53*	.62**	-.15	.97***	-										
7. F0 Offset	.30	.26	.36	-.28	.51*	.33	-									
8. F0 G. S.	.38	.15	.23	.98***	-.19	-.12	-.26	-								
9. F0 M. S.	.47*	.22	.33	.92***	-.15	-.11	-.15	.98***	-							
10. Intensity	-.05	.21	.10	-.27	.60**	.57*	.22	-.29	-.31	-						
11. F1	.30	.21	.17	-.06	-.05	-.22	.43	.05	.23	-.29	-					
12. F2	.06	.23	-.09	.19	-.12	-.23	.24	.10	.14	-.09	.34	-				
13. B1	.20	.29	.42	-.05	.54*	.59**	.03	.01	.04	.46*	-.04	-.07	-			
14. B2	-.13	.21	.13	-.25	.49*	.49*	.01	-.26	-.28	.89***	-.33	-.22	.46*	-		
15. B3	.34	.38	.53*	-.16	.56*	.48*	.51*	-.06	.07	.34	.47*	.17	.80***	.28	-	
16. B4	.36	.36	.47*	-.25	.70**	.59**	.67**	-.18	-.08	.47*	.38	.09	.70**	.30	.91***	-

*Note*. AB: adult bilinguals. CB: child bilinguals. EM: English monolingual adults. P. A.: perceptual accuracy. F0 Avg.: F0 average. F0 G. S.: F0 general slope. F0 M. S.: F0 major slope.

***p < .001.

** *p* < .01.

* *p* < .05.

As shown in Table 5, there were three significant acoustic correlates of adult bilinguals’ tone perception: average F0 (*r* = .51), F0 onset (*r* = .49) and F0 major slope (*r* = .47). For child bilinguals, the two significant acoustic correlates were average F0 (*r* = .56) and F0 onset (*r* = .53). For adult English monolinguals, there were four significant acoustic correlates: average F0 (*r* = .62), F0 onset (*r* = .62), B3 (*r* = .53) and B4 (*r* = .47).

Also, there were significant correlations among various acoustic parameters. As shown in Table 5, duration was strongly correlated with F0 general slope (*r* = .98) and F0 major slope (*r* = .92). Average F0 was significantly correlated with F0 onset (*r* = .97), F0 offset (*r* = .51), intensity (*r* = .60), B1 (*r* = .54), B2 (*r* = .49), B3 (*r* = .56) and B4 (*r* = .70). F0 onset was also significantly correlated with intensity (*r* = .57), B1 (*r* = .59), B2 (*r* = .49), B3 (*r* = .48) and B4 (*r* = .59). F0 offset significantly correlated with B3 (*r* = .51) and B4 (*r* = .67). F0 general slope strongly correlated with F0 major slope (*r* = .98). Intensity was moderately significantly correlated with B1 (*r* = .46), B2 (*r* = .89) and B4 (*r* = .47). F1 was significantly correlated with B3 (*r* = .47). B1 significantly correlated with B2 (*r* = .46), B3 (*r* = .80) and B4 (*r* = .70). There was also strong correlation between B3 and B4 (*r* = .91).

## Discussion

The present study demonstrates that, although Cantonese tone and English stress differ in their acoustic composition and linguistic function, both are perceived equally well by Cantonese-English bilingual children who simultaneously learned one tone language (Cantonese) and one stress language (English). In contrast, Cantonese-English bilingual adults and English monolingual adults exhibited superior performance in perceiving English stress than in perceiving Cantonese tone, with the bilingual adults tending to outperform the monolingual adults in both tone and stress perception. Cantonese tone perception and English stress perception were significantly associated with each other in Cantonese-English bilingual children and English monolingual adults, but not in bilingual adults. The high rising (25)-low rising (23) contrast was perceived with the lowest accuracy among all 15 Cantonese tone contrasts across the three groups; acoustic analyses indicate that the high rising (25) and low-rising (23) tones are similar in F0 onset and in average F0 but they differ in their major slopes. The correlation analyses between tone perception performance and acoustic correlates showed that average F0 and F0 onset were associated with tone performance in all groups, but F0 major slope was only associated with tone perception in the bilingual adult group. The correlation analyses also showed that English monolinguals’ sensitivity to spectral balance was significantly associated with their Cantonese tone performance. These results are discussed in terms of the mechanism employed by bilinguals in the perception of tone and stress.

### Dynamic Interactive Processing of Tone and Stress in Cantonese-English Bilinguals

There are at least two possible pathways by which Cantonese-English bilinguals may perceive the two suprasegmental features of their languages, i.e., tone and stress. One is that tone and stress are processed independently by two separate systems. This view is primarily based on the important differences in how tone and stress are assigned. As described in the introduction, although the phonetic realizations of Cantonese tone and English stress both involve the manipulation of vocal pitch, there are striking differences between Cantonese tone and English stress. First, tone involves variation in pitch at the syllable level whereas English stress is related to vocal pitch, duration and intensity between syllables [18]. Second, Cantonese tone is used to distinguish a broad set of words in Cantonese, while only a limited subset of content words are distinguished by stress in English [52]. Additionally, the rate of fluctuation in fundamental frequency is higher in tone marking than in stress marking [53]. These differences raise the possibility that tone and stress are processed independently. The alternative possibility is that tone and stress are interdependently and interactively processed within a single perceptual system [54,55].

If tone and stress are indeed processed independently, we would not observe any significant correlation between tone perception and stress perception in bilinguals. Nor would we observe a developmental difference between children and adults in tone and stress perception, given the existing evidence that 10-year-old children perform as well as native adults in tone perception [31] and stress perception [32]. Importantly, we would also not expect any facilitative effect of bilingualism when processing suprasegmental features of language, which we saw, for example, in the fact that Cantonese-English bilingual adults outperformed English monolingual adults on stress perception. Therefore, our results do not support the view that tone and stress are processed fully independently.

Instead, our results support the claim that tone and stress are interdependently and interactively processed by Cantonese-English bilingual children and adults, at least in the acoustic-phonetic processing stage. This view does not completely deny the existence of some independent processing for tone and stress. Rather, we suggest that Cantonese-English bilinguals may have a common suprasegmental representation that embodies the shared acoustic cues relevant to both tone and stress (i.e., average F0 and F0 onset), as well as separate representations that characterize tone-specific cues (e.g., major slope) and stress-specific cues (e.g., spectral balance) respectively. Thus, as an utterance unfolds, both the common suprasegmental representation and the relevant language-specific suprasegmental representations are activated simultaneously. This activation causes no competition between tone and stress processing, resulting in the observed comparable and correlated performance in Cantonese-English bilingual children. As we will discuss below, we found differences between bilingual adults and children in the present study; however, we suggest that this might be accounted for by the possibility that use of suprasegmental representations, both common and language-specific, are constrained by one’s level of language experience and phonological processing capabilities. This reflects the dynamic nature of suprasegmental speech processing.

Our view that tone and stress are processed interactively is consistent with models of second language perception [1,2,56–58]. According to these models, a listener’s perceptual strategy arises from the interaction between their linguistic knowledge and their speech perception system. Specifically, listeners’ linguistic knowledge imposes considerable demands on their speech perception system; the system either assimilates novel sounds into existing categories or alters its structure to accommodate the novel speech sound. Moreover, these assimilation and accommodation processes are constrained by features of listeners’ native languages [1,59].

Of relevance to the present study, our group of Cantonese-English bilingual children grew up in highly bilingual families and in a bilingual society. Cantonese lexical tones and English lexical stress are both present in and permeate their daily language environment. Such extensive dual-language input must have an impact on their speech perception systems, as well as on the phonetic perception of prosodic cues. Our acoustic analyses show that certain acoustic correlates are common across tone and stress, i.e., average F0 and F0 onset. Thus, it seems very likely that Cantonese-English bilingual children developed a common suprasegmental representation that records those language-general acoustic correlates and makes them available for both tone and stress processing. In other words, the concurrent learning of two different types of suprasegmental features leads to the formation of a general suprasegmental prototype that is used for both Cantonese and English. Meanwhile, our analyses also showed that some acoustic correlates are only available to tone or stress processing, such as major slope for tone and spectral balance for stress; this results in the development of separate tone-specific and stress-specific representations in bilingual children.

Whereas bilingual children showed comparable performance on tone and stress perception, both bilingual and monolingual adults exhibited superior performance in perceiving stress than in perceiving tone. This was to be expected for the English monolingual adults, as they are exposed to stress-based English but not tonal Cantonese. However, the finding was surprising for the bilingual adults, in that Cantonese-English bilingual adults had better perceptual performance on non-native English stress than native Cantonese tone. Below, we will discuss the possibility that different task demands contributed in part to this finding, but it is important to note that the bilingual adults tended to perform significantly better on both tone contrast perception test and stress perception test than English monolingual adults. Taken together, these findings with bilingual adults are consistent with a dynamic interactive processing view of tone and stress, suggesting that interdependence between the two may create a facilitative interaction between Cantonese tone and English stress that results from the shared underlying factors that link them together. We argue that Cantonese-English bilingual adults’ stress perception performance being stronger than that of English monolingual adults may be partly due to their having learned to perceive complex tone contrasts, a skill that transfers to and benefits stress perception. Our results imply that learning complex prosodic perception skills, such as those used for Cantonese tones, can generalize to processing simpler prosodic contrasts, such as English stress patterns.

In contrast, it seems unlikely that learning the simple prosodic contrasts of English stress generalize to complex prosodic processing of Cantonese tones. As evident in the results of our acoustic analyses, only a restricted set of acoustic correlates relevant to F0 (i.e., average F0, F0 onset, major slope) are critical to distinguishing tone contrasts. On the other hand, there are many acoustic correlates that can be used to distinguish English stress, i.e., average F0, F0 onset, duration and spectral balance. In other words, many of the tone-related acoustic correlates can be used to distinguish stress, but not all stress-related correlates are helpful to tone perception. Thus, the higher stress perception performance that we see in bilingual adults could be due to the additive effects of the language-general suprasegmental cues, such as F0 average, and the stress-specific cues. We note that in our study, bilingual adults’ tone perception accuracy was not correlated with their stress perception accuracy, although this can be explained by their ceiling performance in both tone and stress. In posing this explanation, we are certainly aware that the present study focuses on perception of Cantonese tone and English stress at the acoustic-phonetic level. Future research is needed to test this explanation by examining suprasegmental speech processing at the lexical level.

While our explanation for the facilitative effect of Cantonese tone on English stress perception may account for bilingual adults’ superior performance on stress perception than tone perception, we must also consider why bilingual children performed similarly on the two tasks. We suggest that although bilinguals have both a common suprasegmental representation and separate tone- and stress-specific representations, the effective use of these representations may be constrained by the bilingual’s level of language knowledge and their acoustic-phonetic processing capabilities. Bilingual adults have extensive linguistic knowledge of Cantonese tones and English stress patterns, affording them more opportunity to develop a sophisticated understanding of the acoustic correlates of tone and stress. In contrast, young bilingual children have only recently acquired all six Cantonese tones and English stress contrasts [31], As noted in the introduction, a very recent study showed that young children's suprasegmental speech perception (specifically English lexical stress) was not as good as that of skilled adults, which can be attributed to children’s comparatively limited language experience [28]. Moreover, individuals’ acoustic-phonetic processing and auditory capabilities mature with age [29,30].

Another likely possibility is that the common suprasegmental representation posited by the interactive account of suprasegmental processing is primarily used during the earliest stages of perceptual development for tone and stress, yielding comparable tone and stress perception performance in our bilingual children, with the separate representations of tone and stress being strengthened over time through language learning. Note that our child participants were balanced Cantonese-English bilinguals who had received balanced input in both languages and whose parents rated them as having equal proficiency in both Cantonese and English. In contrast, our adult participants were Cantonese-dominant bilinguals who rated themselves as being more proficient in Cantonese than in English, despite their early exposure to English (around the age of 3). Given the evidence showing that different amounts of language input from bilinguals’ two languages modulates the perception of lexical stress patterns [60], it is very likely that tone-specific and stress-specific representations are strengthened in bilingual adults through extensive language inputs, leading to the development of separate perceptual strategies to distinguish tone and stress. That is, Cantonese-dominant bilingual adults use F0-related acoustic correlates as the primary cue to perceive tone contrasts, whereas they use stress-related acoustic correlates, such as spectral balance, to perceive stress contrasts. In this way, they are able to achieve a relatively high level of performance in both tone and stress perception.

Although bilingual adults outperformed monolingual adults on stress and tone perception, we observed an asymmetric pattern of performance favouring stress in both groups of adults. This may be due to differences in the relative perceptual salience of tone and stress, as well as in part to differences in perceptual load. In the stress perception test, there were two patterns of consonant-vowel bisyllabic stimuli: either strong stress on the first syllable followed by weaker stress on the second syllable (e.g., SUla), or weak stress on the first syllable followed by strong stress on the second syllable (e.g., suLA). These two patterns resulted in one minimal pair differing only in stress location (e.g., SUla vs. suLA) (see Fig 1B). Our acoustic analyses showed that each stressed syllable tends to have higher frequency and longer duration than the unstressed syllables. By contrast, in the tone perception test, there were six contrastive tones, and they combined into 15 tonal contrasts that varied in pitch height, pitch contour, or both. Moreover, the relative perceptual saliency of some tone contrasts is very low, for example, high rising (25, T2)-low rising (23, T5) (see Fig 1A). Thus, it seems that the task difficulty of the English stress perception test is relatively less, and stress patterns are relatively more predictable, than is the case for the Cantonese tone perception test. However, as noted earlier, the finding that Cantonese-English bilingual adults responded more accurately and more quickly than English monolingual adults to both Cantonese tone and English stress perceptions suggests that task difficulty alone cannot explain superior performance on stress perception compared to tone in bilingual adults. Rather, the facilitative interaction between Cantonese tone and English stress (as discussed above) is responsible for this effect.

The significant perceptual differences observed between bilingual children and adults also suggest that bilingual children’s perceptual abilities in tone and stress improve even after the age of 11 years old. This finding is partially consistent with previous work showing that, by the age of 10, Cantonese children achieved adult-like accuracy in tone perception [31]. Our finding also shows that Cantonese-English bilingual children performed as well as bilingual adults on most, but not all, tone contrasts (e.g., T2-T5 performance was lower). Nevertheless, we must be cautious of the possibility that these developmental differences could be partly due to the effect of bilingualism on tone perception, wherein dual-language exposure to one tone language and one stress language may impact the perceptual organization of suprasegmental speech categories at the early stage of processing. Specifically, unlike Cantonese-dominant children in previous research [31], our balanced Cantonese-English bilingual children have to negotiate the differences between tone and stress, and to characterize two types of prosodic regularities in both of their native languages. The interaction between tone and stress could be facilitative, as we observed in bilingual adults’ superior performance on stress perception. However, it could be also inhibitory at the early stage of processing, for the processing of complex suprasegmental contrasts like Cantonese tones in particular. The possible inhibitory effects of bilingualism on Cantonese tone perception need to be further investigated in future research.

### Acoustic Correlates of Cantonese Tone and English Stress

Another important contribution of the present study is that we systematically analyzed all possible acoustic correlates that could potentially contribute to variability in the perceptual saliency of English lexical stress patterns and of all 15 tone contrasts for the six Cantonese tones. Our acoustic analyses of Cantonese tone suggested that average F0, F0 onset and major slope are the critical acoustic correlates in determining the relative ease of Cantonese tone perception. Cantonese-English bilingual children, adults, and monolingual English-speaking adults could all pick up on average F0 as a cue to discriminate tones. However, when average F0 and F0 onset are similar, as between T2 and T5 in particular, these similarities cause the three groups difficulty in perceiving tone contrasts.

The magnitude of major slopes of the six tones, in descending order, were: T2 > T5 > T3 > T6 = T1 > T4, implying that the perception of the T2-T5 contrast depended on the F0 major slope (in the rising portion). Our perceptual results showed that bilingual adult’s accuracy in distinguishing the T2-T5 contrast was significantly higher than that of bilingual children and English monolingual adults, therefore further supporting that bilingual adults were much more sensitive to F0 major slope.

Although duration, intensity, the formant frequencies F1 and F2, and spectral balance were not critical in accounting for tones’ perceptual saliency, we noticed that English monolingual adults appeared to use spectral balance as a perceptual cue to perceive Cantonese tones. This was indicated by the signification correlations of English monolingual adults’ tone perception performance with spectral energy across bands 1, 3 and 4. Also, English monolingual adults performed worse than both bilingual adults and bilingual children in the T1-T3 contrast alone; recall that only T1 and T3 shared the same spectral energy across the first three bands. This further reinforced our inference that spectral balance was only a predominant cue for perceiving Cantonese tones among English monolingual adults.

The results of our acoustic analyses of English stress patterning suggested that average F0, F0 onset, F0 offset, F0 general slope, duration, intensity, and spectral balance are important acoustic cues to distinguish between stress patterns. These findings are consistent with previous research suggesting that English lexical stress is a multidimensional construct involving F0, duration, and intensity [15,16]. More importantly, we have demonstrated that among all of these cues, spectral balance may be one of the most reliable acoustic correlates of English stress.

### Implications, Limitations and Future Directions

The present study provides compelling evidence, from both a perceptual experiment and detailed acoustic analyses, for the interactive processing of Cantonese tone and English stress in bilinguals. Specifically, the study provides the most comprehensive analyses available to date of acoustic correlates of tone and stress, enabling us to make inferences about the possible perceptual cues employed by bilingual and monolingual listeners in perceiving tone and stress. More importantly, the present study proposes an interactive account of suprasegmental speech processing to account for the performance differences in Cantonese tone and English stress observed among Cantonese-English bilingual children, adults, and English monolingual adults, as well as the acoustic-perceptual associations of stress and tone. Our interactive account of suprasegmental speech processing extends the interactive account of speech perception [61] to suprasegmental speech perception by putting forward the possibility of a facilitative interaction between Cantonese tone and English stress.

However, we should note that the interactive account assumes that bilinguals may build a common suprasegmental representation that encodes both Cantonese tone and English stress. We must be cautious that the common representation assumption is derived from perceptual performance differences between bilinguals and monolinguals, as well as the correlations between Cantonese tone and English stress. We are aware that the perceptual correlation seen here could be due to the fact that there is an underlying common factor that ties Cantonese tone and English stress together, such as the sensitivity to the common acoustic cues (i.e., F0). Thus, it is important for future research to verify whether the perceptual correlation between Cantonese tone and English stress reflects a common suprasegmental representation or the general sensitivity to acoustic cues; studies might do so by adding a perceptual control condition (e.g., perception of other contrasts that do not exist in either Cantonese or English) and examining whether perception of novel contrasts is correlated with tone or stress.

It should also be noted that our tone and stress stimuli were pseudoword CVCV sequences without any lexical meaning. This limits the current study from finding any lexical expectation effects, which have been seen in previous research [11]. It would be worthwhile for future work to consider using real words as perceptual stimuli in order to examine how lexical level information may impact perception of tone and stress. The AXB discrimination task has limitations due to the way in which it has participants classify stimuli. It could be that participants made their “different” or “same” decisions on the basis of auditory sensitivity instead of linguistic information. Like previous tone perception research, we used 400ms as the inter-trial interval to allow the auditory features of the previous trial’s stimuli to fade. However, the question of how fast auditory memory fades, or even if it fades at all, is debatable [62,63]. Additionally, the AXB design may place significant demands on working memory/attention resources, which may underlie individual differences. In particular, our adult participants may have more stability in executive function than the children. Thus, future research might consider taking steps to address these potential limitations. For example, including non-linguistic pitch stimuli would help to determine whether the pattern of results obtained for non-linguistic pitch might be similar to what we found here. Varying the inter-trial interval would also be worthwhile as a way to examine the effects of inter-trial interval on perception of suprasegmental speech. Including working memory and auditory attention measures in future work will enable researchers to remove the possible effect of individual differences in working memory and executive function on performance on an AXB discrimination task. Such research would provide further insight into the perceptual mechanisms employed by bilinguals in perceiving tone and stress.

Nevertheless, our results add to the existing body of work on cross-language speech perception. We show that, despite their differences on acoustic and linguistic dimensions, tone and stress are processed interactively in Cantonese-English bilinguals. Moreover, the relative ease of tone perception is determined by average F0, F0 onset, and F0 major slope. On the other hand, distinguishing stress patterns relies on multiple acoustic correlates, including spectral balance, F0-related features, duration, and intensity. The most robust acoustic correlate of English stress is spectral balance. Listeners’ prevalent difficulty in perceiving the high rising (T2)-low rising (T5) tone contrast arises from their similarities in F0 onset and average F0, and the primary cue to distinguish this contrast is F0 major slope. These findings emphasize the interactive processing mechanism of suprasegmental speech perception, and also suggest a role of one’s level of linguistic knowledge in the development of perceptual strategies used in speech perception.
